# Lactobacillus cell envelope-coated nanoparticles for antibiotic delivery against cariogenic biofilm and dental caries

**DOI:** 10.1186/s12951-022-01563-x

**Published:** 2022-08-02

**Authors:** Luting Weng, Lang Wu, Rongjuan Guo, Jiajia Ye, Wen Liang, Wei Wu, Liang Chen, Deqin Yang

**Affiliations:** 1 Stomatological Hospital of Chongqing Medical University, No. 426, Songshi North Road, Yubei District, Chongqing, 401147 China; 2 Chongqing Key Laboratory of Oral Diseases and Biomedical Sciences, Chongqing, 401147 China; 3 Chongqing Municipal Key Laboratory of Oral Biomedical Engineering of Higher Education, Chongqing, 401147 China; 4 Bioengineering College of Chongqing University, No.174 Shazhengjie, Shapingba, Chongqing, 400044 China

**Keywords:** *Streptococcus mutans*, *Lactobacillus*, Nanoparticles, Cariogenic biofilm, Cell membrane

## Abstract

**Background:**

Due to their prevalence, dental caries ranks first among all diseases endangering human health. Therefore, the prevention of caries is of great significance, as caries have become a serious public health problem worldwide. Currently, using nanoscale drug delivery systems to prevent caries has received increased attention. However, the preventive efficacy of these systems is substantially limited due to the unique physiological structure of cariogenic biofilms. Thus, novel strategies aimed at combating cariogenic biofilms to improve preventive efficiency against caries are meaningful and very necessary. Herein, inspired by cell membrane coating technology and *Lactobacillus* strains, we coated triclosan (TCS)-loaded poly(lactic-co-glycolic acid) (PLGA) nanoparticles (TCS@PLGA-NPs) with an envelope of *Lactobacillus* (LA/TCS@PLGA-NPs) and investigated their potential as a nanoparticle delivery system against cariogenic biofilms and dental caries.

**Results:**

LA/TCS@PLGA-NPs were successfully prepared with favorable properties, including a coated envelope, controllable size, negative charge, sustained drug-release kinetics and so on. The LA/TCS@PLGA-NPs inherited native properties from the source cell surface, thus the LA/TCS@PLGA-NPs adhered to *S. mutans*, integrated into the *S. mutans* biofilm, and interfered with the biofilm formation of *S. mutans*. The nanoparticles significantly inhibited the activity, biomass and virulence gene expression of *S. mutans* biofilms in vitro. Additionally, LA/TCS@PLGA-NPs exhibited a long-lasting inhibitory effect on the progression of caries in vivo. The safety performance of the nanoparticles is also favorable.

**Conclusions:**

Our findings reveal that the antibiofilm effect of LA/TCS@PLGA-NPs relies not only on the inheritance of native properties from the *Lactobacillus* cell surface but also on the inhibitory effect on the activity, biomass and virulence of *S. mutans* biofilms. Thus, these nanoparticles could be considered feasible candidates for a new class of effective drug delivery systems for the prevention of caries. Furthermore, this work provides new insights into cell membrane coating technology and presents a novel strategy to combat bacterial biofilms and associated infections.

**Graphical Abstract:**

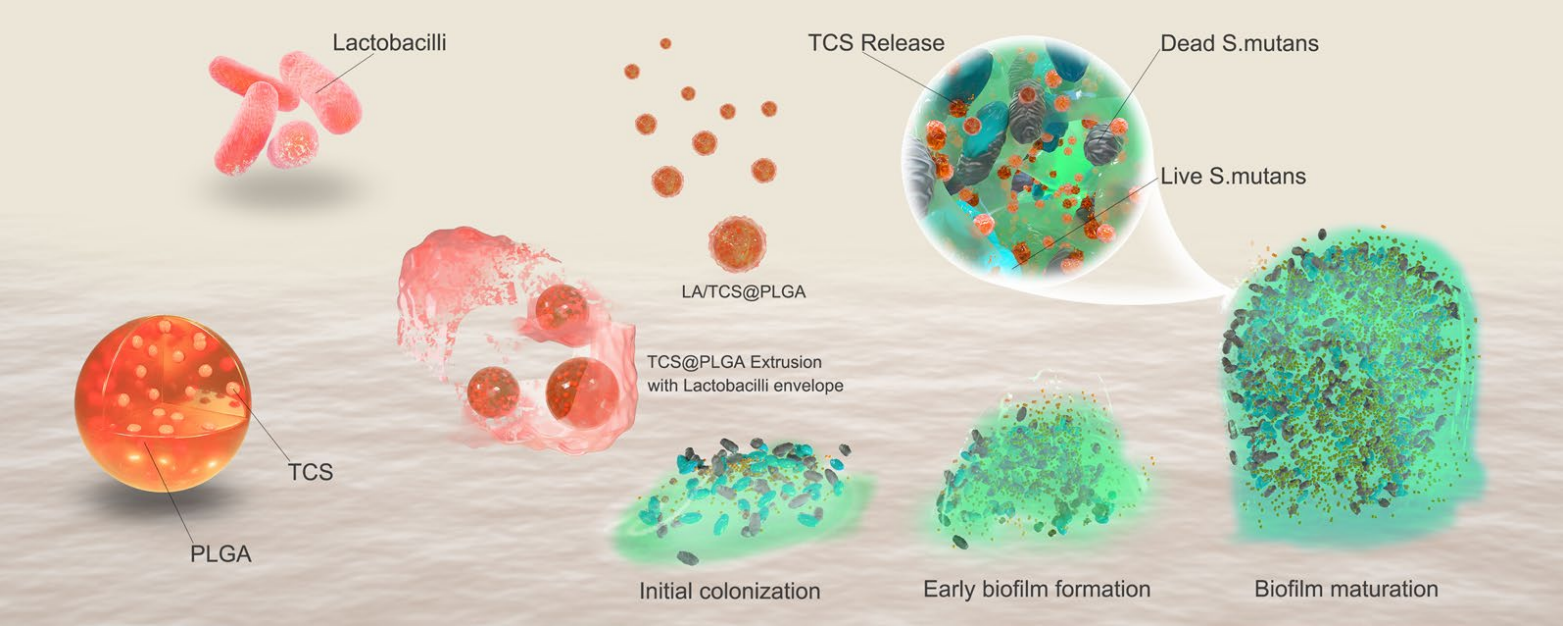

**Supplementary Information:**

The online version contains supplementary material available at 10.1186/s12951-022-01563-x.

## Background

Dental caries is a chronic, progressive, and destructive disease that is mainly caused by bacterial infection. Caries are characterized by a high incidence and prevalence, low treatment rate, wide distribution and irreversible damage. If untreated, caries may lead to the spread of infection to the periapical area of the tooth and beyond [1]. A report from the Global Burden of Diseases, Injury and Risk Factors Study (GBD) 2016 showed that the prevalence of dental caries in permanent teeth was 2.44 billion; thus, caries rank first in all diseases endangering human health [2]. Caries have become a serious public health problem worldwide. The prevention of caries is of great significance.

Dental caries are mainly caused by cariogenic biofilms. Despite significant advances in the prevention of dental caries, cariogenic biofilms are challenging to treat because bacterial pathogens in a mature biofilm, such as *Streptococcus mutans* (*S. mutans*), live in a self-produced matrix of extracellular polymeric substances (EPS), making them difficult to reach by topical antimicrobial agents [3]. Furthermore, it is difficult to retain antimicrobial agents in the biofilm matrix due to the dynamic environment in the mouth and the agents can be deactivated once inside a biofilm by binding to matrix components or through enzymatic modification [4]. Therefore, the penetration and retention of antimicrobial agents into biofilms are key considerations in designing strategies against cariogenic biofilms.

To overcome these hurdles, nanoscale drug delivery systems can be used to control biofilms and prevent caries; thus, these systems have received increased attention in recent years [5, 6]. These delivery systems provide several advantages, including enhanced penetration into the biofilm due to their small size, controlled release behavior and increased stability. For example, mesoporous silica nanoparticles encapsulated with pure chlorhexidine (CHX) with an average particle diameter of approximately 140 nm were demonstrated to have promising antimicrobial effects against planktonic *S. mutans*, monospecies and multispecies bacterial biofilms [7]. Carboxyl-terminated poly(amido amine) (PAMAM-COOH) dendrimers were synthesized and loaded with triclosan, which could bind with the tooth surface to induce in situ remineralization and simultaneously release antibacterial agents for local treatment [8]. Triclosan-loaded tooth-binding micelles prepared by alendronate-modified pluronics (ALN-P) exhibit a high affinity for the hydroxyapatite surface, which could prevent *S. mutans* biofilm accumulation on the exposed tooth surface and reduce the viability of preformed *S. mutans* biofilms [9]. Liposomes prepared by cationic lipids with a mean particle size of approximately 180 nm had high affinity for planktonic *S. mutans* and were able to penetrate the deep layers of *S. mutans* biofilms [10]. Although many delivery systems have been developed, the synthetic strategies involved in preparing those delivery systems are relatively complex and costly, especially for large-scale manufacturing, which limits their wide applications. Moreover, as more antibacterial functionalities are needed, the application of synthetic strategies becomes increasingly difficult and impractical.

More recently, a novel nanoparticle delivery system, cell membrane-coated nanoparticles (CMCNPs), has gained much interest [11]. CMCNPs are generally fabricated by coating the outer membrane of a source cell directly onto the surface of a synthetic nanoparticle. As a result, the complexity of the cell membrane can be almost completely preserved on the surface of the CMCNPs, which endows the CMCNPs with cell-mimicking properties. Although the mechanism remains unclear, it was proposed that the surface molecules on cell membranes and the physicochemical properties of nanoparticles could play major roles in the formation of CMCNPs [12, 13]. Various cell membranes have been employed for coatings thus far, such as erythrocytes, white blood cells, platelets, mesenchymal stem cells, cancer cells and bacterial cells [14]. Using this top-down strategy, CMCNPs not only exhibit advantages as nanocarriers but also preserve the complex functions of source cell membranes, including the ability to interact with other cells. For example, PLGA nanoparticles coated with plasma membranes of *H. pylori* bacteria bear the same surface antigens as the source cells and thus possess inherent abilities of adhering to gastric epithelial cells [15]*.* PLGA nanoparticles coated with a human platelet membrane have several inherent platelet properties, including an ability to bind *Staphylococcus aureus*, which expresses a serine-rich adhesin for platelets [16]. PLGA nanoparticles coated with human MDA-MB-435 cancer cells preserved the homologous targeting ability of the source cell and thus had a much higher affinity toward MDA-MB-435 cells than bare PLGA nanoparticles [17]. Therefore, cell membrane coating nanotechnology breaks through the limitations of traditional surface modification of nanocarriers and has promising potential to exploit the natural properties of source cells for effective drug delivery.

*Lactobacillus* strains are among the most studied and consumed probiotic bacteria, are generally regarded as safe (GRAS) and have been used for a long time in foods and dietary supplements. Recent studies have confirmed that *Lactobacillus* strains, even if heat-killed, can inhibit the colonization and biofilm formation of *S. mutans* in the oral cavity [18–20]. It was suggested that the inhibitory effect is associated with the coaggregation between *Lactobacillus* strains and *S. mutans*. Although the coaggregation mechanism remains largely unknown, the cell surface components of *Lactobacillus* strains, such as peptidoglycan, teichoic acids, polysaccharides, and proteins, are suggested to play a major role in the adhesion of *Lactobacillus* strains to *S. mutans* [21–23].

Therefore, inspired by cell membrane coating technology and *Lactobacillus* strains, we aimed to utilize the cell envelope of *Lactobacillus* strains to coat synthetic polymeric nanoparticles loaded with antimicrobial agents and investigate their potential as a nanoparticle delivery system against cariogenic biofilms. We hypothesized that by inheriting the native properties of the cell surface of *Lactobacillus* strains, this nanoparticle delivery system can (1) adhere to *S. mutans*; (2) interfere with the biofilm formation of *S. mutans*; and (3) integrate in the biofilm as a depot for sustained drug release to inhibit *S. mutans* biofilms.

To test this hypothesis, triclosan (TCS) was chosen as the drug to be incorporated into the nanoparticles. TCS is a synthetic, chlorinated phenolic broad-spectrum antibiotic that is particularly effective against gram-positive microorganisms [24]. It is weakly soluble in water but dissolves well in most organic solvents. TCS has been widely used in oral care products such as toothpastes and mouthwashes, since it was approved by the FDA in 1997. Studies have demonstrated that TCS has a significant inhibitory effect on *S. mutans* [25–28]. Although the mechanism remains unclear, it was proposed that TCS could inhibit the membrane-bond F-ATPases, the phosphoenolpyruvate phosphotransferase system (PTS) and other glycolytic enzymes in *S. mutans* to decrease the cariogenicity of *S. mutans* in suspensions and biofilms [28]. In addition, *S. mutans* was chosen for the present study because it is considered a major etiological agent of human dental caries [29]. *S. mutans* is a Gram-positive facultative anaerobic bacterium with a thick cell wall that consists of many layers of peptidoglycan, which surround the cytoplasmic membrane. Other glycopolymers, such as teichoic acids or polysaccharides, and surface proteins, such as glucosyltransferase (GTF), are embedded within the peptidoglycan layers [30]. It is well established that *S. mutans* has a biofilm-dependent lifestyle. The formation of biofilms helps *S. mutans* resist external adverse factors, promotes synergy between bacteria and forms a quorum sensing system so that the bacteria could experience stronger environmental adaptability in biofilms. Studies have shown that *S. mutans* was 100 to 1000 times more resistant to antibiotics in the biofilm state than in the planktonic state. Therefore, both in vivo and in vitro *S. mutans* biofilm models have been widely used to evaluate the effect of substances on cariogenic biofilms and dental caries [31, 32]. Finally, poly(lactic-co-glycolic acid) (PLGA) was selected as the drug carrier. PLGA is a synthetic biodegradable polymer in the medical field and was approved by the US Food and Drugs Administration and European Medicine Agency. Biocompatibility, biodegradability, flexibility, and minimal side effects are the main advantages when using this polymer for biomedical applications. In the field of stomatology, PLGA is mainly used as the delivery carrier of antibacterial drugs and bioactive molecules and has received continuous attention from scholars [33].

**Scheme 1:**
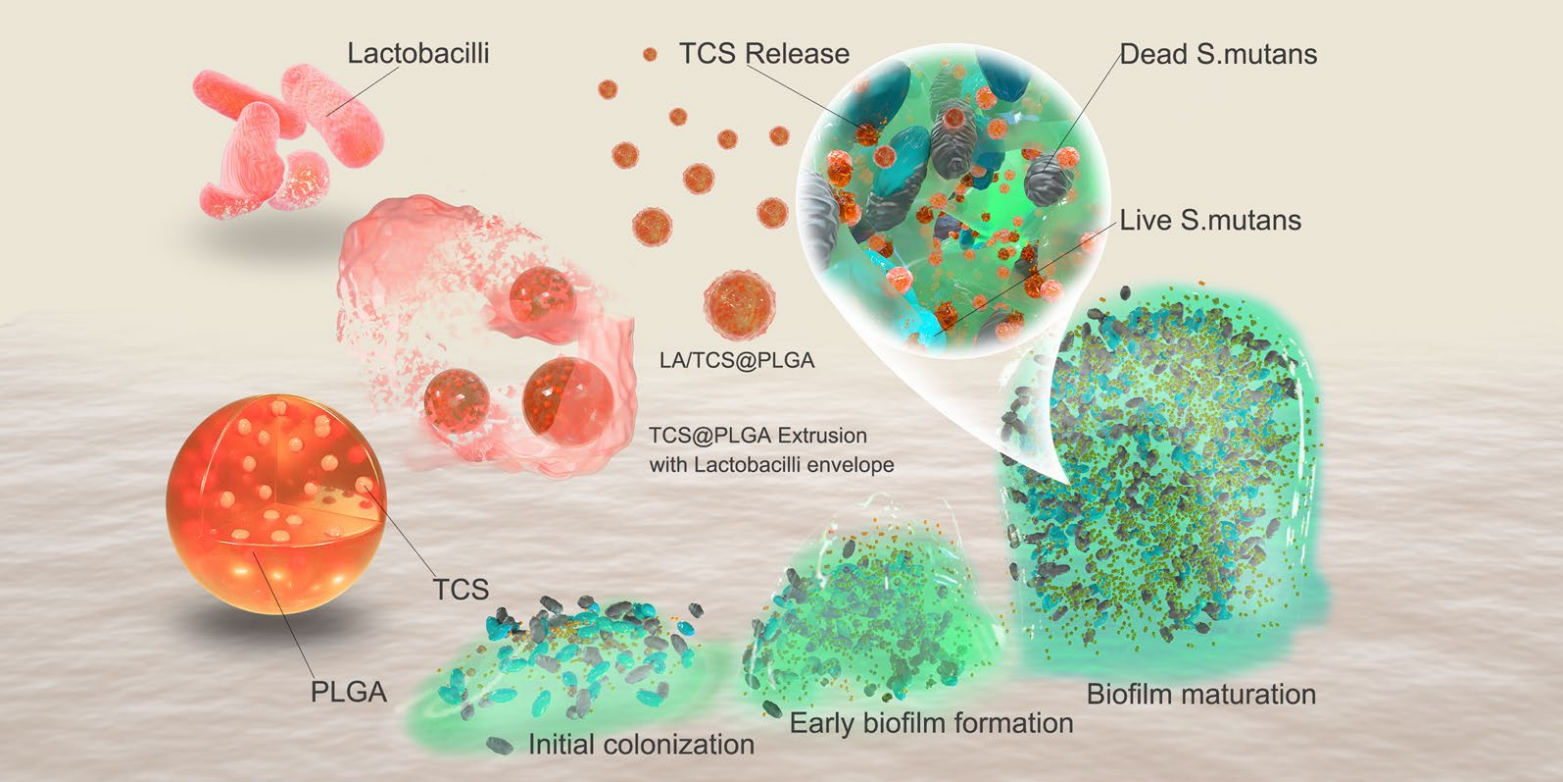
Schematic illustrations displaying the preparation of LA/TCS@PLGA-NPs for interference with *S. mutans* biofilm development

In this study, we loaded poly(lactic-co-glycolic acid) nanoparticles with triclosan (TCS@PLGA-NPs). These TCS@PLGA-NPs are further coated with the cell envelope of *Lactobacillus acidophilus* (*L. acidophilus*). *L. acidophilus* is one of the most widely used probiotics and has the capacity to adhere to *S. mutans* [34]. We demonstrate that the resulting nanoparticles (LA/TCS@PLGA-NPs) inherit surface proteins from the source *L. acidophilus*. The nanoparticles can bind with *S. mutans*, interfere with *S. mutans* biofilms and integrate into the biofilm as a depot for sustained drug release. Treatment with LA/TCS@PLGA-NPs can effectively prevent and inhibit the biofilm formation of *S. mutans* and reduce the caries-associated virulence factors of *S. mutans* biofilms. In addition, LA/TCS@PLGA-NPs exhibit a long-lasting inhibitory effect on the progression of caries in vivo. Overall, this strategy presents a novel strategy to combat bacterial biofilms and associated infections (Scheme 1).

## Results and discussion

### Fabrication and characterization of LA/TCS@PLGA-NPs

**Fig. 1:**
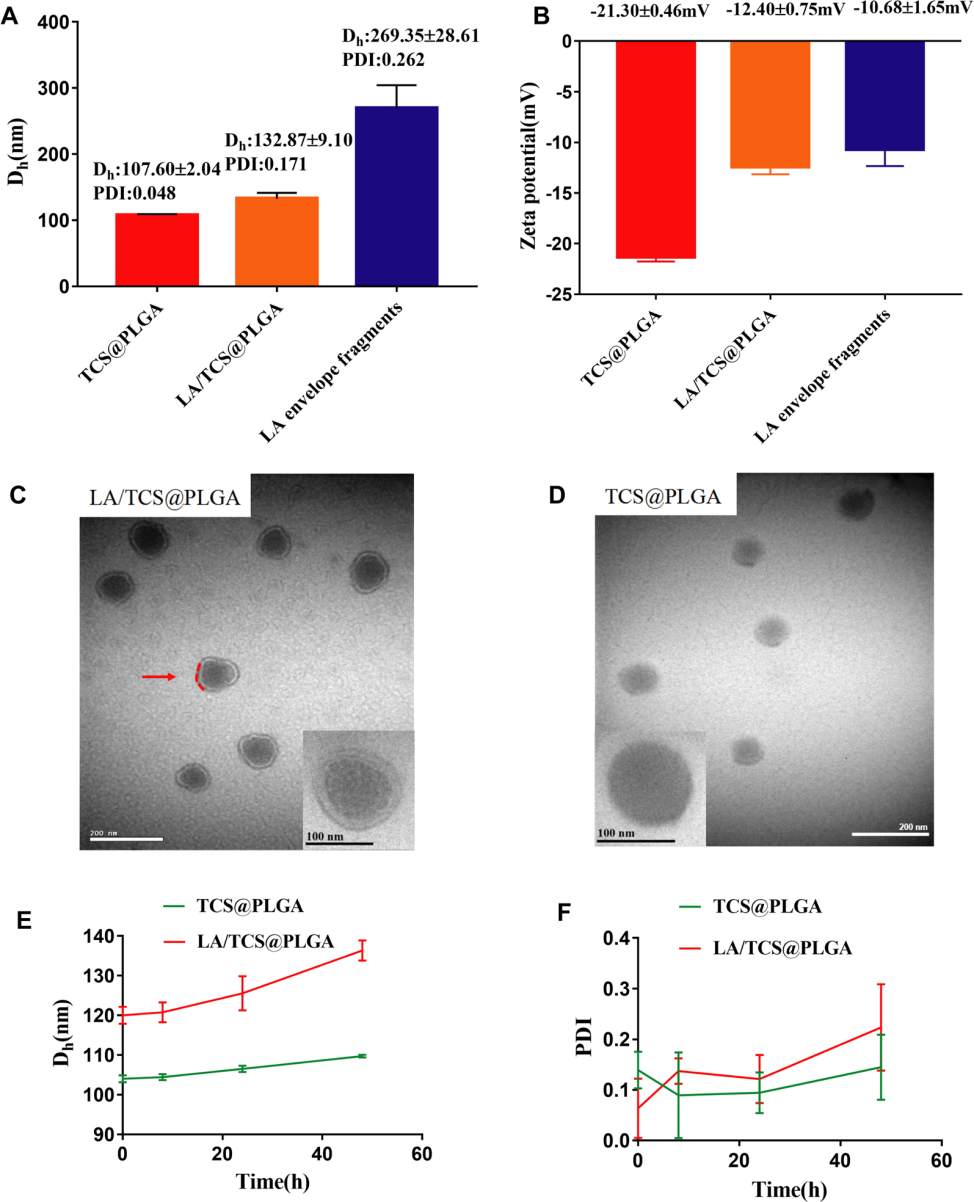
Fabrication and characterization of LA/TCS@PLGA-NPs. **A** The Dh and **B** zeta potential of TCS@PLGA-NPs, LA/TCS@PLGA-NPs and *L. acidophilus* envelope fragments (n = 3, mean ± SD). TEM images of **C** LA/TCS@PLGA-NPs and **D** TCS@PLGA-NPs (scale bar = 200 nm; inside, the red dashed circles represent the cell envelope). The inserts are the enlarged details (scale bar = 100 nm). **E** The diameters and **F** PDI of TCS@PLGA-NPs and LA/TCS@PLGA-NPs in PBS over a span of 48 h. The data points are reported as the mean ± standard deviation

The *L. acidophilus* envelope fragments and TCS@PLGA-NPs were fused to prepare LA/TCS@PLGA nanoparticles (LA/TCS@PLGA-NPs) by an extrusion method [35]. The measurement of LA/TCS@PLGA-NPs with dynamic light scattering showed a hydrodynamic diameter of 132.8 ± 9.1 nm. The diameter increased by approximately 30 nm compared to that of the bare TCS@PLGA-NPs (107.60 ± 2.04 nm), consistent with the addition of a cell envelope onto the nanoparticle surface [36, 37]. Despite the fact that the diameter of envelope fragments was 269.35 ± 28.61 nm, the diameter of LA/TCS@PLGA was close to that of TCS@PLGA-NPs, suggesting that the envelope was wrapped around the TCS@PLGA-NPs (Fig. 1A). In addition, the surface zeta potential of the nanoparticles increased from − 21.3 ± 0.46 mV for TCS@PLGA-NPs to − 12.40 ± 0.75 mV for LA/TCS@PLGA-NPs, which was similar to that of envelope fragments (− 10.68 ± 1.65 mV) (Fig. 1B). The increase in the zeta potential indicated the charge screening effect conferred by the coated envelope [38]. We further analyzed the morphologies of LA/TCS@PLGA-NPs and TCS@PLGA-NPs using TEM. Both LA/TCS@PLGA-NPs (Fig. 1C) and TCS@PLGA-NPs (Fig. 1D) showed a uniform spherical morphology. LA/TCS@PLGA-NPs displayed a clear envelope surrounding the sphere of TCS@PLGA-NPs. Overall, these results demonstrated that the *L. acidophilus* envelope was successfully coated onto TCS@PLGA-NPs.

Moreover, LA/TCS@PLGA-NPs showed a relatively constant hydrodynamic diameter after 48 h of storage at 4 °C, indicating their favorable stability properties (Fig. 1E, F). This result is consistent with a previous study on bacterial membrane-coated nanoparticles [39]. However, compared with PLGA-NPs, the stability of LA/TCS@PLGA-NPs decreased after coating. Although the mechanism for this phenomenon is unknown, we speculated that it is related to the surface properties of the LA envelope, which was inherited from *L. acidophilus*. It has been reported in the literature that *L. acidophilus* exhibits autoaggregation, which is the bacterium–bacterium adhesion of identical strains [40, 41]. Autoaggregation is generally mediated by self-recognizing surface structures of bacteria, such as proteins and exopolysaccharides [42]. Therefore, we proposed that by inheriting the surface properties of *L. acidophilus*, LA/TCS@PLGA-NPs may have an autoaggregating ability, which make the stability of the nanoparticles less than that of PLGA-NPs in this experiment.

### Characterization of proteins

**Fig. 2:**
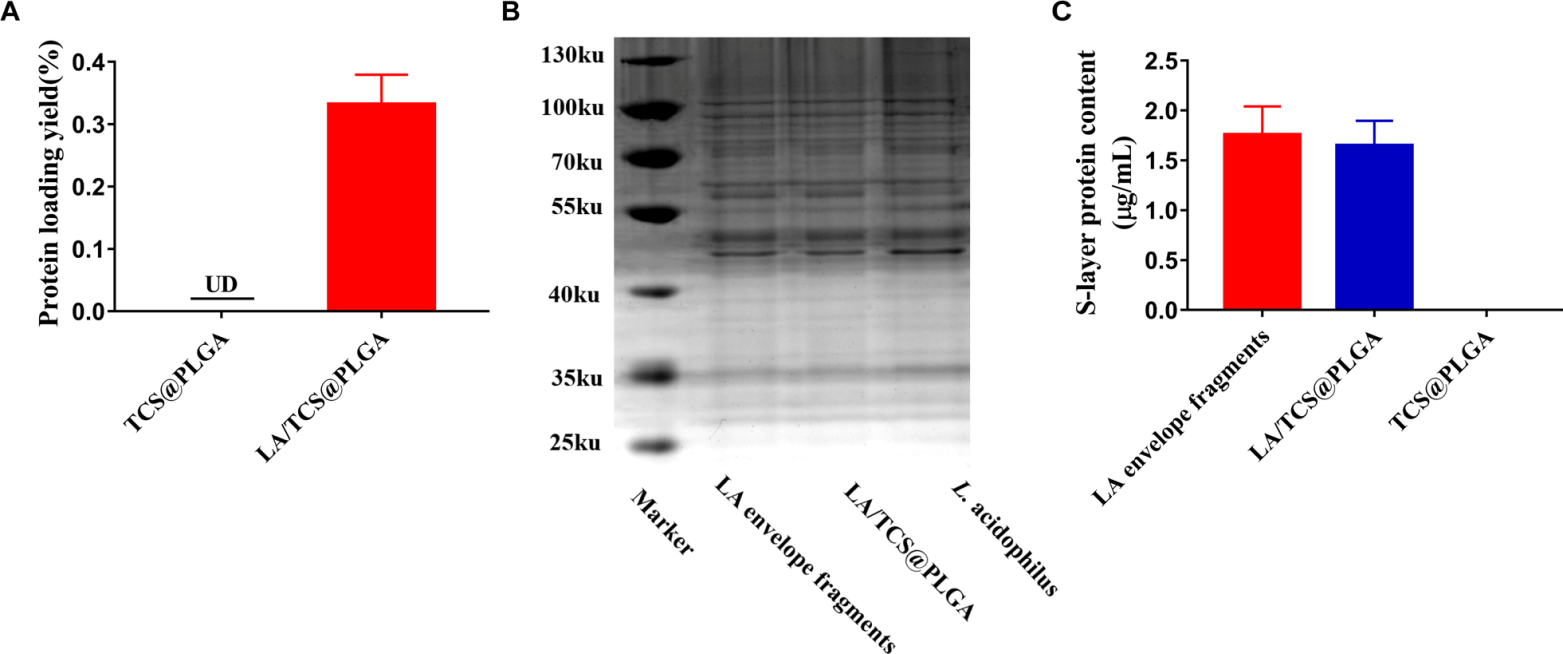
Characterization of proteins. **A** Quantification of the protein content on LA/TCS@PLGA-NPs. Error bars represent standard deviations (n = 3). **B** SDS–PAGE protein characterization of LA envelope fragments, LA/TCS@PLGA-NPs and *L. acidophilus*. **C** Comparison of the S-layer protein content recovered from equivalent amounts of LA envelope fragments, LA/TCS@PLGA-NPs and TCS@PLGA-NPs after LiCl solution treatment (n = 3)

Accumulating evidence in the literature suggests that cell surface proteins are critical for the adhesion ability of *L. acidophilus*[22, 23]. Thus, we examined whether LA/TCS@PLGA-NPs maintained the cell surface proteins of *L. acidophilus*. The surface protein content on LA/TCS@PLGA-NPs was quantified with a protein bicinchoninic acid (BCA) assay. While no protein content was detected from bare TCS@PLGA-NPs, LA/TCS@PLGA-NPs showed a clear presence of protein, as indicated by absorption at 562 nm. Further quantification indicated a protein loading yield, defined as the weight ratio between the immobilized proteins and LA/TCS@PLGA-NPs, of approximately 0.33 ± 0.04 wt% (Fig. 2A).

Furthermore, the surface protein of LA/TCS@PLGA-NPs was characterized by sodium dodecyl sulfonate-polyacrylamide gel electrophoresis (SDS–PAGE). The results suggested that *L. acidophilus* envelope fragments and LA/TCS@PLGA-NPs were highly consistent in protein bands, revealing that almost all envelope proteins were retained throughout the LA/TCS@PLGA fabrication (Fig. 2B).

### Identification of the envelope orientation of LA/TCS@PLGA-NPs

Surface layer (S-layer) proteins are an array of single proteins that are noncovalently bound to the outermost cell envelope of *L. acidophilus* and can be extracted by lithium chloride (LiCl) [43]. Owing to the asymmetric distribution of the S-layer protein on the extracellular side of the envelope, the protein can be used as an indicator to quantitatively analyze the envelope sidedness on LA/TCS@PLGA-NPs. Moreover, since the *L. acidophilus* envelope is impermeable to LiCl, LiCl extraction was applied to examine the S-layer protein content on the outer surface of LA/TCS@PLGA [37]. As shown in Fig. 2B, C, S-layer protein (≈ 46 kDa) on the surface of LA/TCS@PLGA-NPs was clearly visible (Fig. 2B), and the average content of S-layer protein on LA/TCS@PLGA-NPs was approximately 93.82% of the equivalent amount in free envelope fragments (Fig. 2C). This quantification suggests that the S-layer protein is strongly retained on the outside surface of the LA/TCS@PLGA-NPs, confirming the “right-side-out” orientation of the *L. acidophilus* envelope on the nanoparticles.

### Drug loading and in vitro drug release of LA/TCS@PLGA-NPs

TCS has been widely used in oral hygiene products. Nevertheless, the extremely low water solubility (10 µg/mL) has significantly restricted its application in the treatment of bacterial infections [44]. Here, we improved its solubility (approximately 5 times) by successfully loading 49.7 µg of TCS into 1 mL of PLGA aqueous solution (1 mg/mL). As shown in Additional file 1: Fig. S1A, the loading efficiency (LE) and encapsulation efficiency (EE) of TCS@PLGA-NPs were 19.1% and 49.7%, respectively. In addition, envelope cloaking TCS@PLGA-NPs did not lead to a sharp drop in the LE and EE of LA/TCS@PLGA-NPs, which were 14.8% and 47.2%, respectively.

The release kinetics of TCS from TCS@PLGA-NPs and LA/TCS@PLGA-NPs were investigated in PBS (pH 7.4) solution to simulate the physiological environment. In the first 6 h, 30.25% and 25.36% of TCS was released quickly from TCS@PLGA-NPs and LA/TCS@PLGA-NPs, respectively. The early sudden release phenomenon may be due to the free TCS that was not wrapped in nanoparticles or attached to the surface of nanoparticles. Then, the speed of release gradually slowed down. After 77 h of incubation in PBS, 80.82% of TCS was released from TCS@PLGA-NPs, and 64.97% was released from LA/TCS@PLGA-NPs (Additional file 1: Fig. S1B). Except at 0 h and 4 h, the cumulative TCS release of LA/TCS@PLGA-NPs was significantly lower than that of TCS@PLGA-NPs at other time points (*P* < 0.05). This result is consistent with previous studies reporting that the PLGA-NPs coated by cell membrane showed a slower drug release profile than uncoated PLGA-NPs [35, 45]. This result indicated that LA/TCS@PLGA-NPs had better sustained release ability than that of TCS@PLGA-NPs, which may be ascribed to the additional cell envelope bilayer acting as a diffusion barrier.

### Cytotoxicity assay

The cytotoxicity of TCS, PLGA-NPs, TCS@PLGA-NPs and LA/TCS@PLGA-NPs at a series of concentrations was evaluated by CCK-8 assay using HOK cells. As Additional file 1: Fig. S2 shows that TCS exhibited low cytotoxicity at concentrations lower than or equal to 7 µg/mL but high cytotoxicity at concentrations higher than 15 µg/mL. This result is consistent with a previous study on the cytotoxicity of TCS [46]. In addition, PLGA-NPs, TCS@ PLGA-NPs and LA/TCS@ PLGA-NPs had high safety performances. The highest concentrations of PLGA, TCS@PLGA and LA/TCS@PLGA in the experiment were all 120 µg/mL. The cell viability of HOK cells treated with PLGA, TCS@PLGA and LA/TCS@PLGA at the highest concentration for 72 h was more than 75%, which were all in the safe range.

### Adhesion of LA/PLGA-NPs to planktonic *S. mutans*

**Fig. 3:**
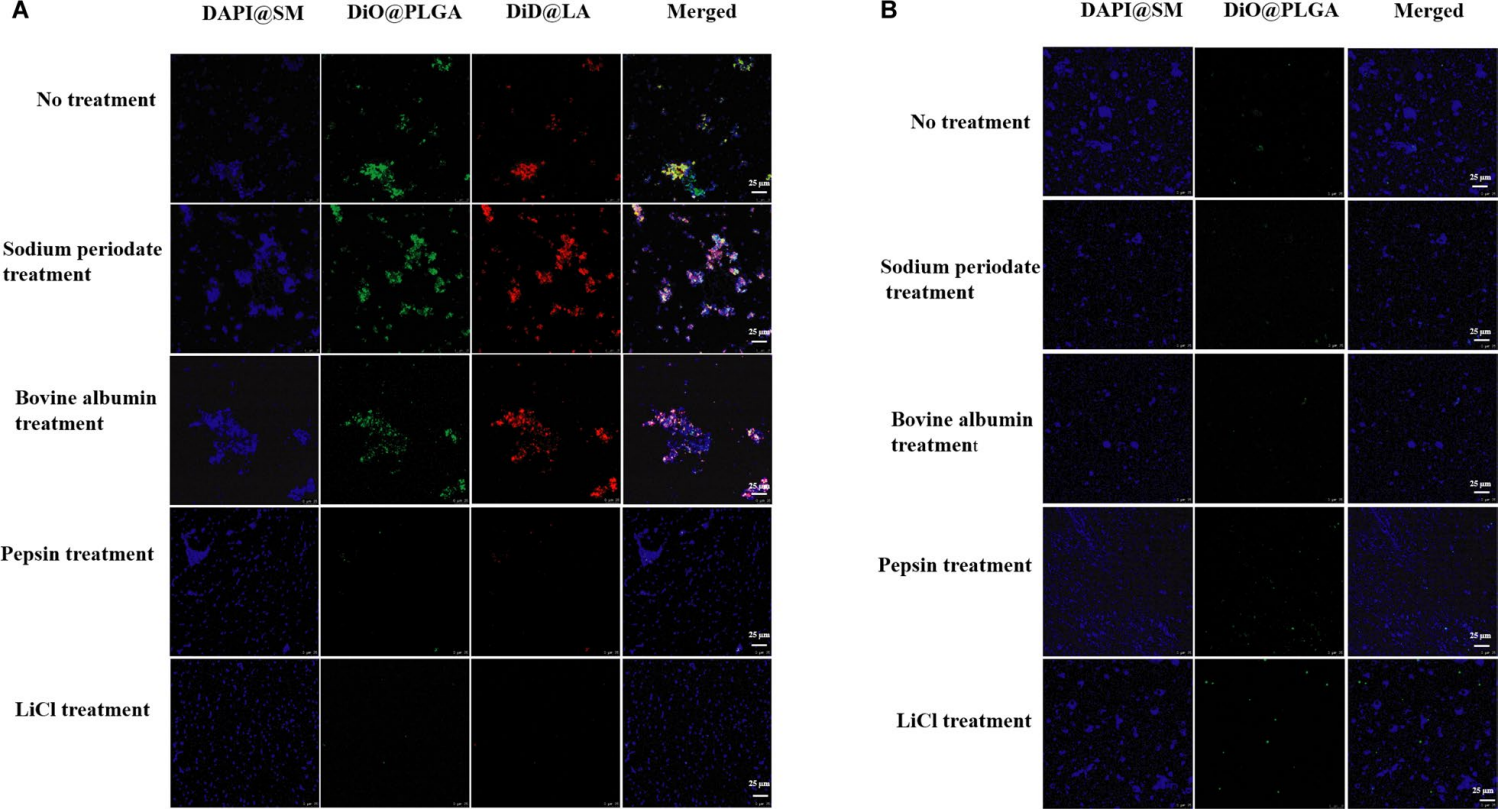
Microscopic assay for the adhesion ability of LA/PLGA-NPs to *S. mutans*. CLSM images for the adhesion of *S. mutans* (blue) with **A** LA/PLGA-NPs (red/green) and **B** PLGA-NPs (green), which were treated with sodium periodate, bovine albumin, pepsin and LiCl. Scale bar = 25 µm

The coaggregation between planktonic *L. acidophilus* and *S. mutans* is shown in Additional file 1: Fig. S3. Based on this information, we hypothesized that the inheritance of native properties of the *L*. *acidophilus* cell surface confers adhesion ability to LA/PLGA-NPs. To test this hypothesis, we labeled LA/PLGA-NPs with fluorescence dye and mixed them with planktonic *S. mutans*. Following incubation and washing, CLSM showed that the LA/PLGA-NPs were retained (green, representing the PLGA-NPs; red, representing the LA envelope) by *S. mutans* (blue), which indicated nanoparticle-cell adhesion (Fig. 3A). In the control group, the fluorescence of PLGA-NPs (green) nearly disappeared after washing (Fig. 3B). The results demonstrated that the *L. acidophilus* envelope could indeed endow PLGA-NPs with the ability adhere to *S. mutans.* Next, we utilized fluorescence spectrophotometry to quantitatively analyze the adhesion ability of LA/PLGA-NPs to *S. mutan*s according to the aggregation assay with slight modifications. As shown in Additional file 1: Fig. S4, the adhesion rate of LA/PLGA-NPs was 66.24 ± 4.27%, which was much higher than that of PLGA-NPs (9.83 ± 2.23%) (*P* < 0.05).

To test the potential adhesin that mediates the adhesion of LA/TCS@PLGA-NPs to *S. mutans*, we employed sodium periodate, bovine serum albumin, LiCl and pepsin to block the adhesion mediated by polysaccharides, teichoic acids and S-layer proteins, respectively [37, 47, 48]. The fluorescence of LA/PLGA-NPs after LiCl and pepsin treatment almost disappeared around *S. mutans*, but the nanoparticles were still retained by *S. mutans* after treatment by sodium periodate and bovine serum albumin (Fig. 3A). The results indicated that the adhesion ability of LA/TCS@PLGA-NPs was polysaccharide- and teichoic acid-independent, and the S-layer protein was probably the major adhesin mediating the adhesion of LA/TCS@PLGA-NPs to *S. mutans*. This result is consistent with previous studies reporting that the S-layer protein plays a key role in the adhesion of *lactobacilli* [22, 49].

### Integration of LA/PLGA-NPs into the *S. mutans* biofilm structure

According to a report by Nobbs [50], the formation period of *S. mutans* biofilms includes the following key stages: 0 h, initial bacterial adherence; 6 h, initial bacterial colonization; 12 h: initial early biofilm formation; 24 h: maturation of early-stage biofilm and 48 h: maturation of the later-stage biofilm. In this study, we added fluorescence-labeled LA/PLGA-NPs at these five stages to interfere with the formation of *S. mutans* biofilms and observed their spatial distribution in *S. mutans* biofilms by CLSM.

**Fig. 4:**
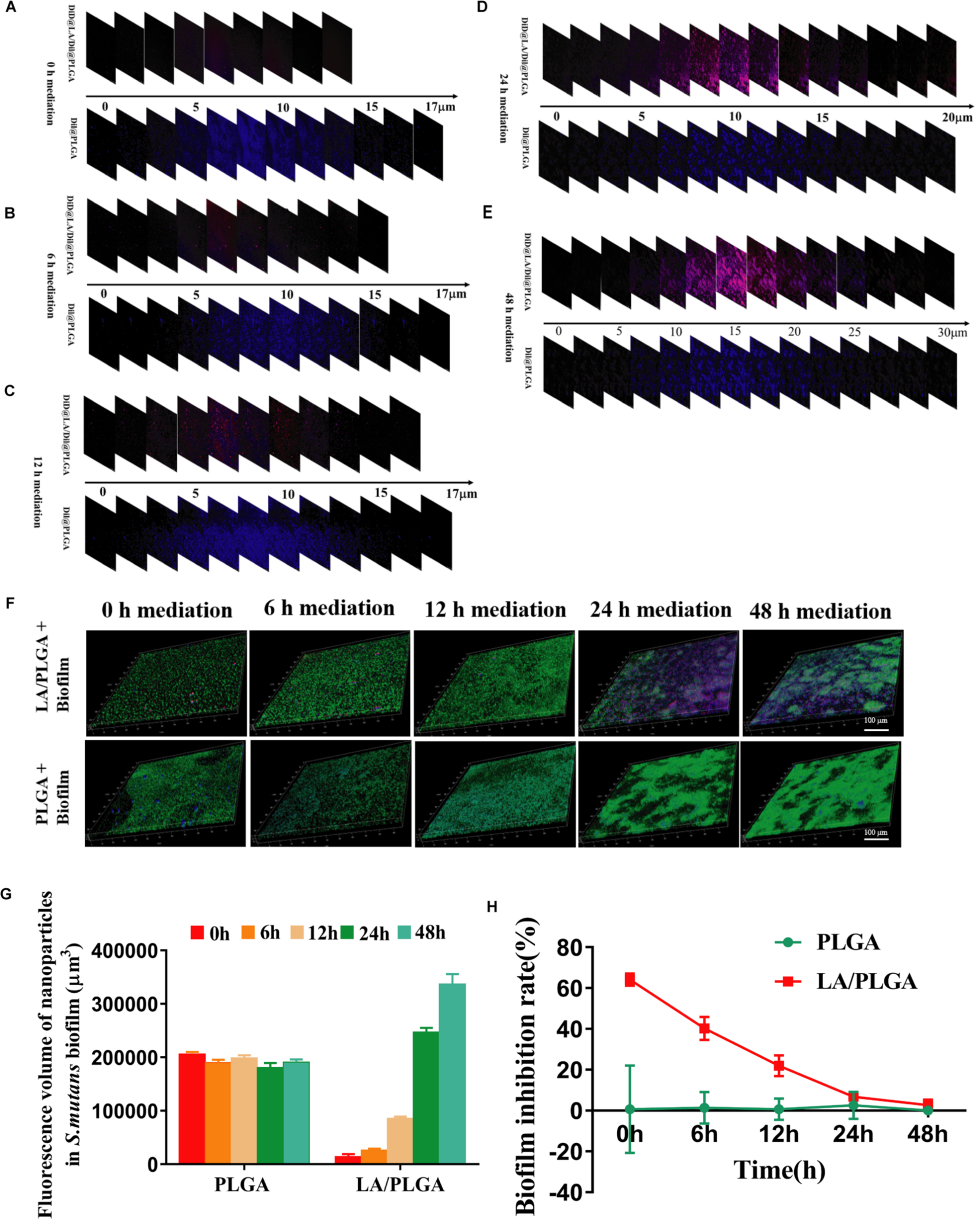
Integration of PLAG-NPs and LA/PLGA-NPs into the *S. mutans* biofilm structure. **A**–**E** CLSM images of the penetration depth of PLAG-NPs (blue) and LA/PLGA-NPs (red/blue) in the biofilms. **F** 3D CLSM images of the spatial distribution of LA/PLGA-NPs (red/blue) and PLGA-NPs (blue) in *S. mutans* (green) biofilms. Scale bar = 100 μm. **G** Quantitative data of the fluorescence volume of PLGA-NPs and LA/PLGA-NPs in *S. mutans* biofilms. **H** Inhibitory effect of LA/PLGA-NPs on *S. mutans* biofilms. All assays were performed three times, and the data are expressed as the mean ± standard error of the mean

Based on 3D CLSM images, we calculated the biofilm thickness using a Leica Application Suite X (Leica Microsystems, Wetzlar, Germany). As shown in Fig. 4A–E, for the biofilms mediated at 0 h, 6 h, and 12 h, the biofilm thicknesses were all approximately 17 µm, while for the biofilms mediated at 24 h and 48 h, the biofilm thicknesses were approximately 20 µm and 30 µm, respectively. Then, the biofilms were evenly divided into three layers based on these calculated results, representing the inner, medium and outer layers.

We discovered that regardless of which biofilm stage the LA/PLGA-NPs and PLGA-NPs were added at, they mostly existed in the medium layer of biofilms (5–15 µm) without being washed out easily. Such a spatial distribution may be related to the unique structure of biofilms, in which the inner and outer layers were loose and dispersed but the medium layer was dense and cross-linked. Additionally, as shown in Fig. 4F and G, the quantity of LA/PLGA-NPs increased with the progress of biofilm formation, while the change trend of PLGA-NPs remained relatively consistent during biofilm formation. Thus, we speculated that the quantity of LA/PLGA-NPs existing in biofilms was closely related to that of *S. mutans*. One possible reason is that LA/PLGA-NPs possessed an ability to adhere with *S. mutans*. Therefore, we proposed that the integration of LA/PLGA-NPs into *S. mutans* biofilms relies not only on their particle size but also on their adhesion to *S. mutans*.

### Interfering Effect of LA/PLGA-NPs on *S. mutans* biofilm formation

To explore whether by inheriting the surface properties of *L. acidophilus*, LA@PLGA-NPs without TCS could interfere with the biofilm formation of *S. mutans*, we further quantitatively analyzed biofilm inhibition by the crystal violet method. Because it was previously reported in literatures that inactivated probiotic strains, such as inactivated *L. acidophilus*, have no antibacterial activity, but can bind with planktonic pathogens through surface properties, and thus block planktonic pathogens from colonization and biofilm formation.

The results showed that at different time points, the biofilm inhibition rates were 64.1%, 40.3%, 21.9%, 6.7% and 2.6%, respectively (Fig. 4H), suggesting that LA/PLGA-NPs mainly inhibited the early stage of biofilm formation. In addition, PLGA-NPs had no significant inhibitory effect on the biofilm of *S. mutans*. Similar results were observed in previous studies on the inhibitory effect of live or heat-killed *Lactobacillus* strains against *S. mutans* biofilms [18, 51–53]. Although the specific mechanism is not clear, we proposed that this effect could be attributed to the surface properties of LA/PLGA-NPs, which were able to coaggregate with planktonic *S. mutans* in the medium solution and thus inhibit biofilm formation at the early stage. However, once the biofilm matures, it is difficult for LA/PLGA-NPs to exclude and displace *S. mutans* in the biofilm.

### Effect of LA/TCS@PLGA-NPs on *S. mutans* biofilm activity

**Fig. 5:**
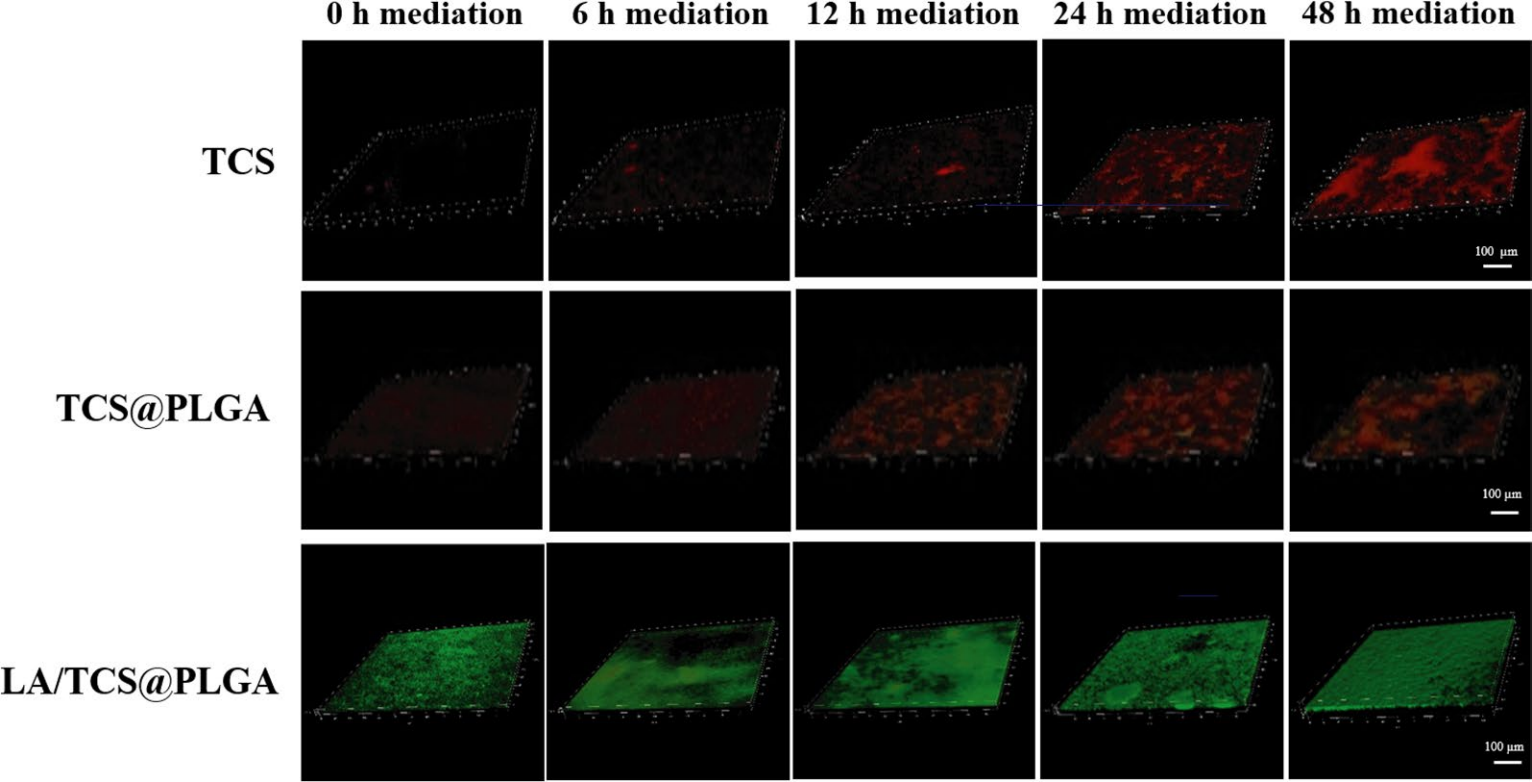
3D CLSM images of *S. mutans* biofilms on the glass coverslips mediated at 0 h, 6 h, 12 h, 24 h, and 48 h and treated with TCS, TCS@PLGA-NPs and LA/TCS@PLGA-NPs (green represents live bacteria; red represents dead bacteria). Scale bar = 100 μm

**Fig. 6:**
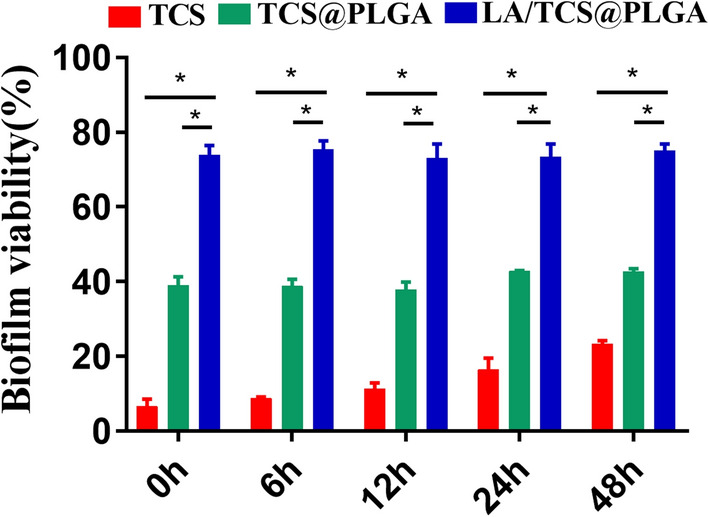
Quantitative data of the *S. mutans* biofilm activity mediated at 0 h, 6 h, 12 h, 24 h, and 48 h and treated with TCS, TCS@PLGA-NPs and LA/TCS@PLGA-NPs. The assay was performed three times, and the data are expressed as the mean ± standard error of the mean. *P < 0.05

In this experiment, the effect of LA/TCS@PLGA-NPs on the biofilm activity was evaluated by a live and dead bacterial staining kit and was observed using CLSM. As shown in Fig. 5, live bacteria appeared as fluorescent green, while dead bacteria appeared as fluorescent red. The biofilms treated with TCS and TCS@PLGA-NPs showed few live bacteria. In contrast, the biofilms treated with LA/TCS@PLGA-NPs had many live bacteria. The intensities of red fluorescence (dead bacteria) and green fluorescence (live bacteria) were quantitatively measured by ImageJ software. Then, the biofilm activity was calculated as the percentage of live bacteria over both live and dead bacteria. The results revealed that compared with the control groups, in which the biofilm activity remained constant at approximately 89%, the activity of biofilms treated with TCS, TCS@PLGA-NPs and LA/TCS@PLGA-NPs was significantly decreased (*P* < 0.05). However, as shown in Fig. 6, the activity of biofilms treated with LA/TCS@PLGA-NPs was significantly higher than that treated with TCS and TCS@PLGA-NPs when nanoparticles were added at any stage of biofilm development (*P* < 0.05). These results indicated that during the short period of time (24 h), LA/TCS@PLGA exhibited less of an inhibitory effect on the biofilm activity of *S. mutans* biofilms than that of TCS and TCS@PLGA-NPs. We proposed that this phenomenon was probably due to the dual barrier effect of LA/TCS@PLGA-NPs. In the biofilms, the cumulative release of TCS from LA/TCS@PLGA-NPs in the short term was less than that of TCS@PLGA-NPs.

Moreover, when TCS and TCS@PLGA-NPs were added at the maturation stage, the biofilm activity was higher than that when they were added at the early stage (*P* < 0.05). This was probably because at the maturation stage, *S. mutans* was embedded in an extracellular matrix of polymeric substances, which had a negative impact on the antibacterial effects of both TCS and TCS@PLGA. However, for LA/TCS@PLGA-NPs, the biofilm activity remained constant at approximately 75% regardless of when the nanoparticles were added (*P* > 0.05). This result indicated that the TCS delivered by LA/TCS@PLGA-NPs was less affected by the biofilm matrix at the maturation stage. We proposed that on the one hand, the presence of a cell envelope and polymeric nanoparticles could protect TCS from being deactivated by matrix components or enzymatic modifications. On the other hand, due to the ability to adhere to with *S. mutans*, LA/TCS@PLGA-NPs could reach and release TCS more closely to *S. mutans* in the biofilm, while TCS released from TCS@PLGA-NPs may be blocked and inactivated by the matrix on the way to *S. mutans*. Thus, even when added at the mature stage, LA/TCS@PLGA-NPs could maintain a consistent inhibitory effect.

### Effect of LA/TCS@PLGA-NPs on the biomass of *S. mutans* biofilms

**Fig. 7:**
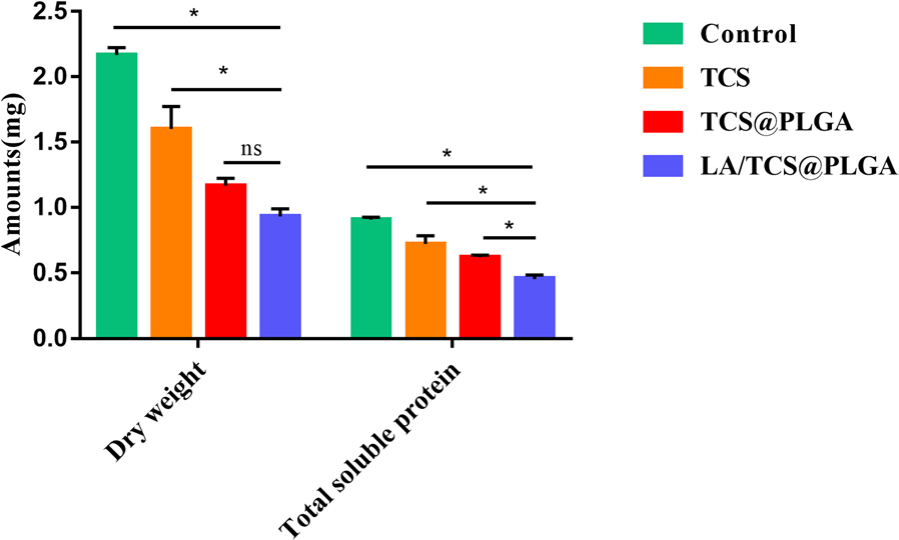
Dry weight and total soluble protein of *S. mutans* biofilm 8 days after the treatment. The data are presented as the mean ± SD, *P < 0.05; ^ns^*P* > 0.05

The biomass growth of the *S. mutans* biofilm was measured from the dry weight and the total soluble protein. As shown in Fig. 7, 8 days after the treatment, the dry weight and total soluble protein of biofilms in the LA/TCS@PLGA-NPs group were 0.93 ± 0.06 mg and 0.46 ± 0.03 mg, respectively, which were the lowest among the different groups, although there was no statistically significant difference in the dry weight of biofilms between the LA/TCS@PLGA-NPs group and TCS@PLGA-NPs group. This result indicated that LA/TCS@PLGA-NPs could effectively inhibit the growth of *S. mutans* biofilms and had a longer-lasting antibiofilm effect than that of TCS and TCS@PLGA-NPs. We speculated that this may be related to the integration of LA/TCS@PLGA-NPs in *S. mutans* biofilms and the cumulative release of TCS from LA/TCS@PLGA-NPs.

### Effect of LA/TCS@PLGA-NPs on virulence gene expression in *S. mutans* biofilms

Bacterial cells in biofilms tend to exhibit biological and phenotypic traits that are extraordinarily distinct from those of their planktonic counterparts, and these traits are accompanied by significant changes in the bacterial gene expression profile [54]. Studies have shown that the cariogenic characteristics of *S. mutans* biofilms are regulated by a variety of genes. In this study, real-time fluorescence quantitative PCR was used to detect and analyze the gene expression regulating the caries-associated virulence factors of *S. mutans* biofilms.

**Fig. 8:**
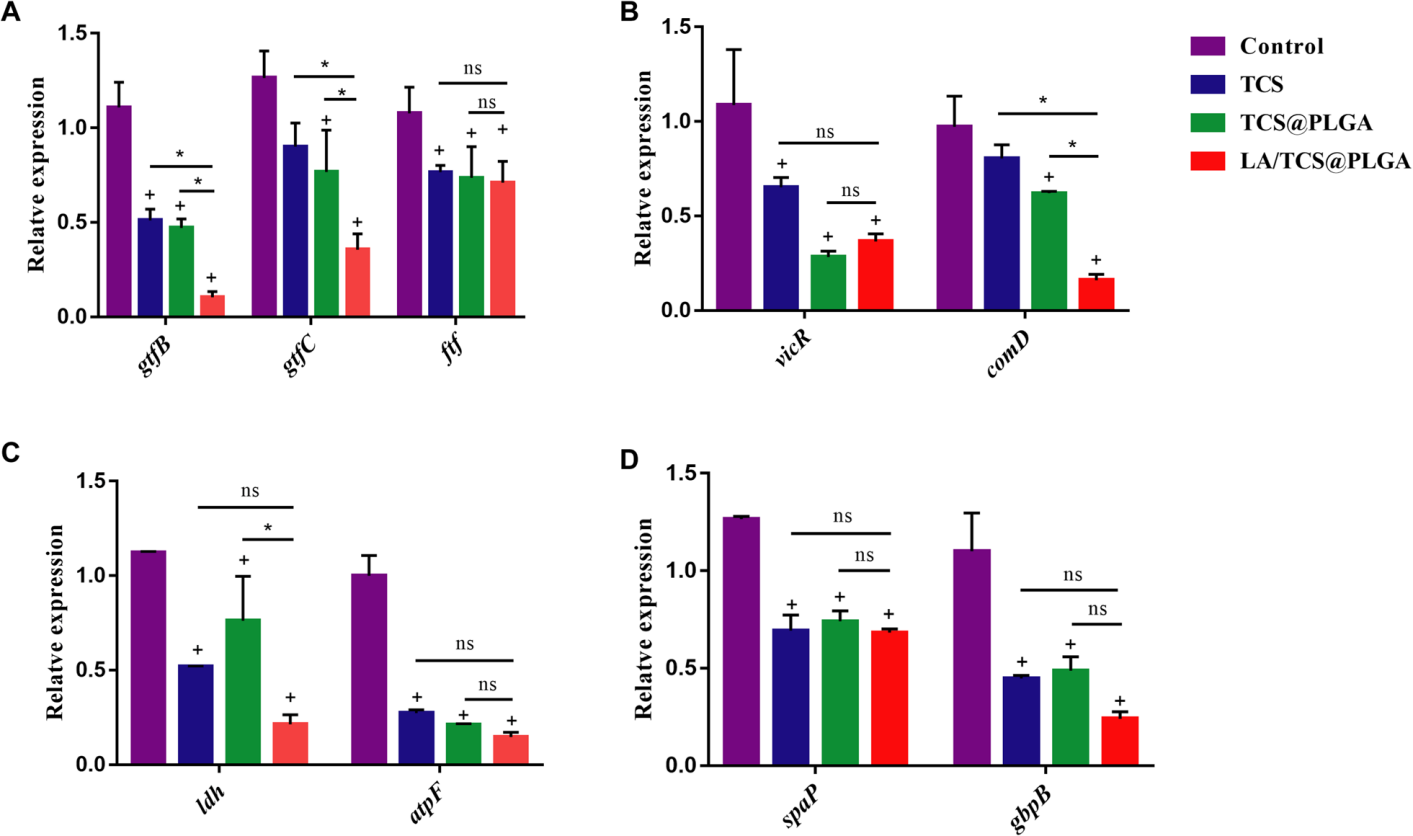
Expression of cariogenicity-related virulence factors of *S. mutans* biofilms by real-time reverse transcription-quantitative PCR. **A** Results for *gtfB*, *gtfC*, and *ftf*. **B** Results for *vicR* and *comD*. **C** Results for *ldh* and *atpF*. **D** Results for *spaP and gbpB*. The level of expression of each gene was normalized to the level of 16S rRNA expression, and the fold change relative to the findings for the control was calculated using the 2^−ΔΔCT^ method. Control, *S. mutans* growth in BHI medium; TCS, *S. mutans* growth in BHI medium supplemented with 5.9 µg/mL TCS; TCS@PLGA, *S. mutans* growth in BHI medium supplemented with 30 µg/mL TCS@PLGA-NPs; LA/TCS@PLGA, *S. mutans* growth in BHI medium supplemented with 40 µg/mL LA/TCS@PLGA-NPs. The results are expressed as the mean ± SD, ^ns^*P* > 0.05; *P < 0.05; + *P* < 0.05, compared with the control group

As revealed in Fig. 8, we found that after a period of 8 days, the expression of caries-associated virulence factors, including *gtfB*, *gtfC*, *fif*, *spaP*, *gbpB*, *ldh*, *atpF*, *comD* and *vicR*, was significantly downregulated in the biofilms treated with LA/TCS@PLGA-NPs compared with that in the control group (*P* < 0.05). In addition, the downregulation of *gtfB*, *gtfC*, and *comD* in response to LA/TCS@PLGA-NPs was significantly greater than that in response to TCS and TCS@PLGA-NPs (*P* < 0.05). Among these genes, *gtfB*, *gtfC*, and *ftf* are related to polysaccharide synthesis and sucrose-dependent adhesion; *gbpB* is related to sucrose-dependent adhesion and biofilm formation; *spaP* is related to sucrose-independent adhesion; *ldh* and *atpF* are related to acid production and acid resistance; and *comD* and *vicR* are related to the stress regulation system [55]. All of these genes are important factors leading to the formation of cariogenic *S. mutans* biofilms. Therefore, these results indicated that the antibiofilm effect of LA/TCS@PLGA-NPs relied not only on the inheritance of native properties of the *L. acidophilus* cell surface but also on the sustained release of antimicrobial agents that inhibit the expression of virulence genes in *S. mutans* biofilms. Based on these results, we proposed that, in terms of virulence gene expression, LA/TCS@PLGA-NPs also exhibited a long-lasting antibiofilm effect, which was better than that of TCS and TCS@PLGA-NPs.

### LA/TCS@PLGA-NPs for dental caries in vivo

To further verify the effectiveness of LA/TCS@PLGA-NPs in vivo, we established a rat model of caries that was induced by the combination of *S. mutans* infection and a cariogenic diet. LA/TCS@PLGA-NPs and other treatments were performed twice daily for 7 consecutive days. After 45 days, the rats were sacrificed. Carious lesions on the smooth surface (Smo) and the sulcal surface (Sul) were quantitatively assessed via Keyes’ scoring [56]. According to Keyes’ scoring, the depth of carious lesions was divided into the following levels: enamel only (E), slightly dentinal (Ds, < 1/4 of the dentin region), moderate dentinal (Dm, 1/4–3/4 of the dentin region), and extensive dentinal (Dx, > 3/4 of the dentin region).

**Fig. 9:**
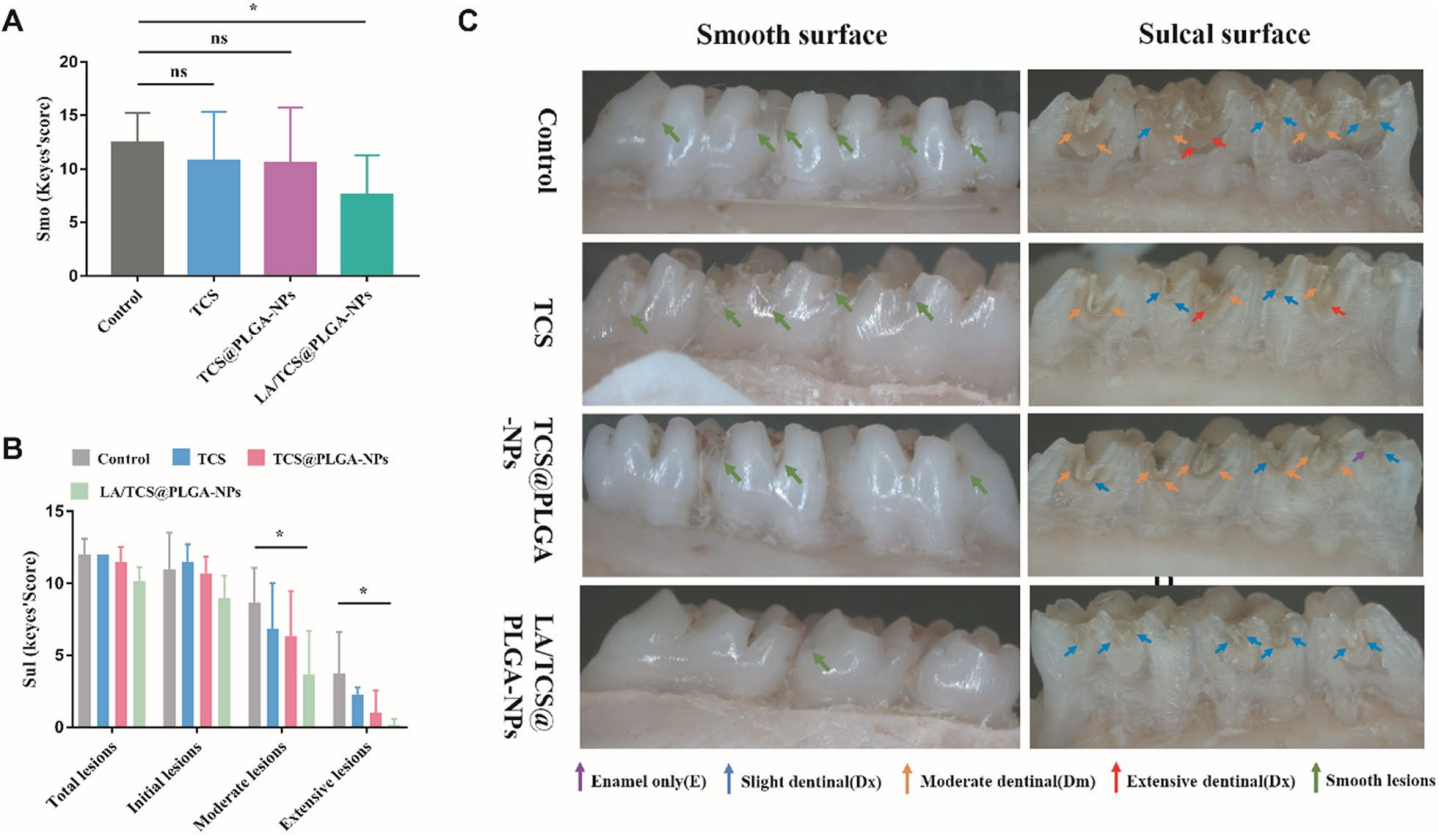
Anti-caries effect after different treatments as evaluated by Keyes’ scoring and the biosafety of LA/TCS@PLGA-NPs in vivo. **A** Quantitative assessment of carious lesions on smooth surfaces. **B** Quantitative assessment of carious lesions on the sulcal surface. **C** Representative images of carious lesions. Data are presented as the mean ± SD. ^ns^*P* > 0.05; *P < 0.05; compared with the control group

For the smooth surface, the carious lesions were evaluated based on the scoring of E. As shown in Fig. 9A, compared with the control group, the carious lesions were significantly decreased in the LA/TCS@PLGA-NPs group (*P* < 0.05), while there were no significant differences between the control group and other treatment groups (*P* > 0.05). For the sulcal surface, the carious lesions were evaluated based on the following levels [56]: total lesions (E + Ds + Dm + Dx), initial lesions (Ds + Dm + Dx), moderate lesions (Dm + Dx), and extensive lesions (Dx). As shown in Fig. 9B, there were no statistically significant differences among the different groups in terms of the total lesions and initial lesions (*P* > 0.05). However, compared with those in the control group, the moderate lesions and extensive lesions in the LA/TCS@PLGA-NP group were significantly decreased (*P* < 0.05), while no significant differences were observed between the control group and other treatment groups. These results indicated that compared with TCS and TCS@PLGA-NPs, LA/TCS@PLGA-NPs exhibited a more lasting inhibitory effect on the progression of caries in vivo. We proposed that this effect could be attributed to the ability of LA/TCS@PLGA-NPs to adhere to *S. mutans* biofilms, which facilitated the retention and sustained drug release of LA/TCS@PLGA-NPs in the oral cavity.

**Fig. 10:**
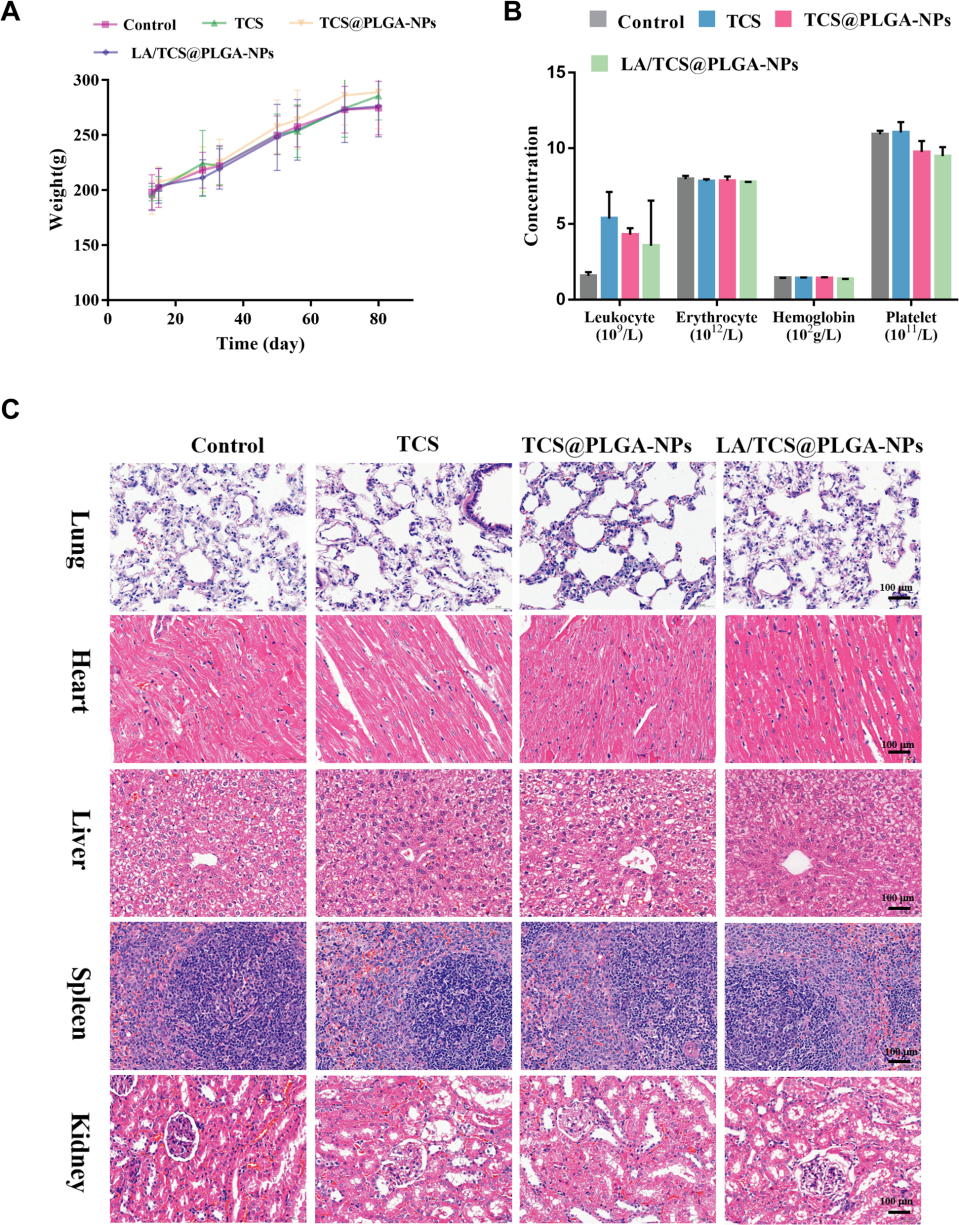
The biosafety of LA/TCS@PLGA-NPs and other treatments in vivo. **A** Body weight changes of rats with different treatments. **B** Routine blood tests, including leukocyte, erythrocyte, hemoglobin and platelet counts. **C** H&E-stained sections of major organs resected from mice subjected to treatment (scale bar = 100 μm)

The in vivo biosafety of LA/TCS@PLGA-NPs was assessed via body weight monitoring, blood biochemical assays, routine blood examination and histological analysis of the main organs. During the treatment period, there was no significant difference in the body weight of the rats among the different groups (Fig. 10). The blood biochemical assays of total cholesterol (TC), triglyceride (TG), high-density lipoprotein (HDL), low density lipoprotein (LDL), immunoglobulin M (IgM), immunoglobulin G (IgG), and glucose (Glu) were at normal levels (Additional file 1: Table S1). Moreover, routine blood examination showed that the levels of leukocytes, erythrocytes, hemoglobin and platelets did not vary significantly between the different groups. In addition, based on the results of hematoxylin–eosin (H&E) staining, no distinguishable change were found in the main organs (heart, liver, spleen, lung, and kidney). Accordingly, these results demonstrated that LA/TCS@PLGA-NPs did not induce significant adverse effects in vivo, indicating that these nanoparticles have potential as a safe treatment for dental caries.

## Materials and methods

### Materials

TCS and PLGA (MW38000-54,000, 50:50) were purchased from Solarbio (Beijing, China). DiD, DiO, Dil and DAPI were purchased from Beyotime (Beijing, China). CFSE was purchased from AbMole (USA). *Lactobacillus acidophilus* strains (ATCC4356) and *Streptococcus mutans* strains (UA159) were provided by the West China Center of Medical Science, Sichuan University. Cell counting kit-8 (CCK-8), human oral keratinocytes, BCA protein assay kit and TRI Reagent were acquired from Sigma–Aldrich (USA). A live and dead bacterial staining kit was purchased from BestiBio Biotechnology Co., Ltd. (Nanjing, China). The SYBRPRIMES qPCR set and Prime Script RT reagent kit were purchased from TaKaRa (Beijing, China). The primers were designed and synthesized by Sangon Biotechnology Co., Ltd. (Shanghai, China).

### Preparation of *L. acidophilus* cell envelope fragments

The *L. acidophilus* cell envelope was prepared according to Tomio [36]. The *L. acidophilus* strains were routinely reactivated in MRS broth at 37 °C under anaerobic conditions overnight. The bacteria were then incubated, harvested at the mid-exponential phase by centrifugation at 5000 × *g* for 10 min, and resuspended in sterile phosphate-buffered saline (PBS) to an optical density (OD) of 0.5 at 600 nm. The resuspended bacteria were ruptured using a freezing grinding device (JINGXIN, China) to extract envelopes. The solution was then centrifuged at 5000 × *g* for 10 min to remove the debris. *L. acidophilus* envelope fragments were recovered as white precipitates after three wash-centrifugation cycles and stored in PBS at 4 °C.

### Preparation of triclosan-loaded PLGA nanoparticles

Triclosan-loaded PLGA nanoparticles (TCS@PLGA-NPs) were prepared through a nanoprecipitation process [57]. Briefly, the weighed amount of PLGA (30 mg) was dissolved in 5 mL acetone together with TCS (3 mg). Five milliliters of this solution was added dropwise into 10 mL of Pluronic^®^ F-68 aqueous solution (0.5%, w/v) under magnetic stirring. Acetone was then left to evaporate at room temperature under magnetic stirring for 4 h. The resulting aqueous dispersion was centrifuged to collect the TCS@PLGA nanoparticles (11,000 rpm, 45 min, 4 °C). After the NPs were collected, they were washed twice with distilled water to obtain purified nanoparticles.

### Preparation of *L. acidophilus* cell envelope-coated TCS@PLGA nanoparticles

The *L. acidophilus* envelope fragments and TCS@PLGA-NPs were fused to prepare LA/TCS@PLGA nanoparticles (LA/TCS@PLGA-NPs) by an extrusion method [35]. Briefly, the *L. acidophilus* envelope fragments and TCS@PLGA-NPs were mixed and extruded 15 times using an Avestin mini-extruder (Avestin, LF-1, Canada) through a 200 nm polycarbonate porous membrane. Following coating, LA/TCS@PLGA-NPs were purified by centrifugation at 10,000 × *g* for 10 min to remove redundant *L. acidophilus* envelope fragments.

### Characterization of nanoparticles

The size and zeta potential of LA/TCS@PLGA-NPs, TCS@PLGA-NPs, and *L. acidophilus* envelope fragments were determined using a Malvern Zetasizer Nano ZS unit (Nano ZS 90, Malvern, UK) with a He–Ne laser (λ = 633 nm) at a scattering angle of 90° at 25 °C. The stability of TCS@PLGA-NPs and LA/TCS@PLGA-NPs was assessed by measuring the size variation of nanoparticles in PBS at 4 °C over 2 days. The morphology of TCS@PLGA-NPs and LA/TCS@PLGA-NPs was visually observed using transmission electron microscopy (TEM) at 200 kV (JEM-2100F, JEOL, Japan).

### Characterization of proteins

The LA/TCS@PLGA-NP protein was characterized by SDS–PAGE. The envelope proteins of *L. acidophilus*, *L. acidophilus* envelope fragments and LA/TCS@PLGA-NPs were extracted by cell total protein extraction kits (Beyotime). The extracted proteins were run on a 12% SDS–PAGE gel in running buffer using a BIO-RAD electrophoresis system at 70 V for 0.5 h and then at 140 V for 1 h. Finally, the SDS–PAGE gel was stained with SimplyBlue for visualization.

The LA/TCS@PLGA-NP protein was further detected by quantitative analysis according to Feng et al. [58]. The LA/TCS@PLGA-NPs were lyophilized and weighed. The protein content of LA/TCS@PLGA-NPs was measured by a BCA protein assay kit. The protein loading yield, which is defined as the weight ratio of immobilized proteins on the nanoparticles, was calculated.

### Identification of the envelope orientation of LA/TCS@PLGA-NPs

Surface layer (S-layer) proteins are an array of single proteins that are noncovalently bound to the outermost cell envelope of *L. acidophilus* and can be extracted by lithium chloride (LiCl) [43]. Owing to the asymmetric distribution of the S-layer protein on the extracellular side of the envelope, the protein can be used as an indicator to quantitatively analyze the envelope sidedness on LA/TCS@PLGA-NPs. Moreover, since the *L. acidophilus* envelope is impermeable to LiCl, LiCl extraction was applied to examine the S-layer protein content on the outer surface of LA/TCS@PLGA [37]. Briefly, LA/TCS@PLGA-NPs were incubated with 5 mol/L LiCl solution at room temperature for 30 min. The samples were centrifuged at 8000 rpm for 15 min. Then, the supernatant was further dialyzed and examined for S-layer proteins by a BCA kit. In this study, the LA/TCS@PLGA-NPs were prepared with excess nanoparticles to ensure that all *L. acidophilus* envelope fragments were occupied. Equivalent amounts of *L. acidophilus* envelope fragments and bare TCS@PLGA-NPs were used as positive and negative controls, respectively.

### Drug loading and in vitro drug release study

To calculate the loading efficiency and encapsulation efficiency, LA/TCS@PLGA-NP and TCS@PLGA-NP lyophilized powders were dissolved in dimethyl sulfoxide (DMSO), and the absorbance was measured by a UV–vis spectrophotometer (DU730, Beckman Coulter) at 290 nm. According to the preestablished standard curve of TCS in DMSO, the LE and EE were calculated using the following equations:

$${\text{LE}}\left( \% \right) = {\text{M}}_{{{\text{TCS}}}} /{\text{M}}_{{{\text{NPs}}}} \times \,100\%$$

$${\text{EE}}\left( \% \right) = \,{\text{M}}_{{{\text{TCS}}}} /{\text{M}}_{{{\text{added}}}} \times 100\%$$

in which M_TCS_ is the mass of TCS loaded in the nanoparticles, M_NPs_ is the mass of TCS@PLGA-NPs or LA/TCS@PLGA-NPs in the formulation and M_added_ is the mass of added TCS.

To examine the drug release profile in vitro, LA/TCS@PLGA-NPs (1 mg/mL, 1 mL) and TCS@PLGA-NPs solutions (1 mg/mL, 1 mL) were added to disposable dialysis bags (MWCO: 38,000 Da) in PBS (10 mL). At different time points, the external drug release buffers were collected, and an equivalent amount of PBS was added. The cumulative amount of TCS released was quantified by a microplate reader.

### Cytotoxicity assay

Cell Counting Kit-8 (CCK-8) assays were performed to measure the cytotoxicity of TCS, PLGA-NPs, TCS@PLGA-NPs and LA/TCS@PLGA-NPs. Human oral keratinocytes (HOKs) were cultured in Dulbecco’s modified Eagle’s medium (DMEM), 100 mg/mL streptomycin, 100 units/mL penicillin and 10% heat-inactivated fetal bovine serum (FBS) at 37 °C with 5% CO_2_ and 95% relative humidity. Then, the cells were seeded in 96-well microtiter plates at a density of 1 × 10^4^ cells/well and incubated in 100 μL of DMEM per well for 24 h. In addition, equivalent amounts of TCS, PLGA PLGA-NPs, lyophilized TCS@PLGA-NPs, and LA/TCS@PLGA-NPs were dissolved in 100 μL cell-grade DMSO and then diluted to serial concentrations with fresh culture media.

The culture media was replaced with fresh culture media containing serial dilutions of TCS, PLGA, TCS@PLGA-NPs and LA/TCS@PLGA-NPs. The cells were incubated for an additional 24 h, 48 h and 72 h. Then, 10 μL of the CCK-8 solution was added to each well and incubated for 4 h. The absorbance was measured using a microplate reader at a wavelength of 450 nm. The cell viability (%) was calculated using the following equations. Cell viability (%) = (M _test_−M_negative control_)/(M_positive control_−M_negative control_) × 100%, in which the M _test_ is the absorbance value of the wells with cells and medicine, the M_negative control_ is the absorbance value of negative wells without cells and medicine and the M_positive control_ is the absorbance value of positive control wells with cells but without medicine.

### Adhesion of LA/PLGA-NPs to planktonic *S. mutans*

We labeled the PLGA-NPs with DiO fluorescence dye (DiO@PLGA-NPs) by a nanoprecipitation process and then coated them with an *L. acidophilus* envelope labeled by DiD dye (DiD@LA/DiO@PLGA-NPs). The nuclei of *S. mutans* were labeled with DAPI (DAPI@SM). After LiCl, pepsin, sodium periodate or bovine albumin treatment, DiO@PLGA-NPs and DiD@LA/DiO@PLGA-NPs were mixed with the DAPI@SM suspension and incubated for 1. The mixed solution was then centrifuged at 3000 × *g* for 10 min to collect sediment. After 3 wash-centrifugation cycles, the sediment was fixed with 4% polyformaldehyde solution for 30 min. The adhesion of nanoparticles to planktonic *S. mutans* was observed by CLSM. The whole process of the experiment was performed in a shaded environment.

To quantitatively analyze the adhesion ability of LA/PLGA-NPs, we prepared DiO@PLGA-NPs (l mg/mL) and DiD@LA/DiO@PLGA-NPs (l mg/mL) and measured their fluorescence intensity using a multifunctional enzyme maker. Then, DiO@PLGA-NPs and DiD@LA/DiO@PLGA-NPs were mixed with the *S. mutans* suspension (1 × 10^8^ CFU/mL, 1 mL) and incubated for 1 h. The mixed solution was centrifuged at 3000 × *g* for 10 min to collect the supernatant. The fluorescence intensity of the supernatant was measured to evaluate the adhesion ability as follows:

$${\text{Adhesion}}{\mkern 1mu} {\text{rate}}\left( \% \right) = \left( {{\text{A}}_{{{\text{Initial}}}} - {\text{A}}_{{{\text{supernatant}}}} /{\text{A}}_{{{\text{Initial}}}} } \right) \times 100\%$$

in which A_supernatant_ is the mass of nanoparticles in the supernatant, and A_Initial_ is the initial mass of nanoparticles.

### Integration of LA/PLGA-NPs into the *S. mutans* biofilm structure

The DiD@LA/Dil@PLGA-NP solution was prepared by the abovementioned method. A biofilm formation assay was conducted as proposed by Loo, with slight modification [59]. Briefly, stationary-phase *S. mutans* were adjusted to an OD600 of approximately 1 and inoculated at a ratio of 1:2 (vol/vol) to the medium solution. An in vitro biofilm model was established using a cover slide as the carrier. Sterile cover slides (18 mm × 18 mm) were placed in sterile 6-well microtiter plates, and 1.5 mL of *S. mutans* suspension was added to each well. The plates were incubated at 37 °C for different periods to promote biofilm formation. After 0, 6, 12, 24, or 48 h of incubation, 100 μL of DiD@LA/Dil@PLGA solution was added to the corresponding well. Afterward, the cultures with 0-, 6-, and 12-h-old *S. mutans* biofilms were incubated for up to a total of 24 h. In addition, the cultures with 24- and 48-h-old *S. mutans* biofilms were incubated for up to a total of 48 and 72 h, respectively. Fresh medium was replaced every 24 h. Dil@PLGA-NPs were prepared and studied as the control group. Finally, the biofilms were rinsed with sterile physiological saline solution 3 times, stained with N01 dyeing liquor, and observed by CLSM.

### Interfering effect of LA/PLGA-NPs on *S. mutans* biofilm formation

To test the interfering effect of LA/PLGA-NPs on the formation of *S. mutans* biofilms, stationary-phase *S. mutans* (50 µL) was diluted twofold in BHI medium (50 µL) and inoculated in 96-well microtiter plates. After 0, 6, 12, 24, or 48 h of incubation, the LA/PLGA-NP solution (0.15 mg/mL, 100 μL) was added to each well and cultured as described above. The medium was replaced with fresh medium every 24 h. Finally, planktonic bacteria were gently aspirated from the microplates. Each well was stained with 200 μL of crystal violet for 30 min at room temperature. After two rinses with saline solution, 100 μL of 95% alcohol was added to release the dye. Biofilm biomass was quantified by measuring the optical density at 600 nm (OD600) in each well using a microplate reader. The control group was treated with the same volume of physiological saline. Biofilm inhibition (%) = 1-(biomass_treatment_/biomass_control_) × 100%

### Effect of LA/TCS@PLGA-NPs on *S. mutans* biofilm activity

The sterile cover slides were placed in sterile 6-well microtiter plates, and 1.5 mL of *S. mutans* suspension was added. After 0, 6, 12, 24, or 48 h of incubation, 10 µL volumes of LA/TCS@PLGA-NPs (6 mg/mL), TCS@PLGA-NPs (4.5 mg/mL) and TCS (0.88 mg/mL) were added individually to the wells, and the cultures were incubated as described above. The medium was replaced with fresh medium every 24 h. Finally, the slides were removed and subjected to fluorescence staining with a live/dead bacterial staining kit. The biofilm structure was observed using CLSM, and Z-stack analysis was performed using Leica Application Suite X (Leica Microsystems, Wetzlar, Germany). The amounts of viable and nonviable bacteria were recorded. Biofilm activity was calculated as the percentage of live bacteria over both live and dead bacteria. All control groups were administered an equivalent volume of physiological saline. In this experiment, the concentrations of LA/TCS@PLGA-NPs, TCS@PLGA-NPs, and TCS in BHI solution (1.5 mL) were 40 µg/mL, 30 µg/mL, and 5.9 µg/mL, respectively. The concentrations of TCS@PLGA-NPs and TCS were calculated based on the concentration of LA/TCS@PLGA-NPs and drug loading, which made the content of TCS from LA/TCS@PLGA-NPs equal to that from TCS@PLGA-NPs and TCS.

### Effect of LA/TCS@PLGA-NPs on the biomass of *S. mutans* biofilms

Sterile cover slides were placed in sterile 6-well plates, to which 1.5 mL of *S. mutans* suspension was added in each well. After 24 h of incubation, 10 µL volumes of LA/TCS@PLGA-NPs (6 mg/mL), TCS@PLGA-NPs (4.5 mg/mL), TCS (0.88 mg/mL) and an equivalent volume of physiological saline were added individually to the wells. The plates were incubated for an additional 24 h. The biofilm was rinsed with physiological saline to remove free TCS and NPs, and the medium was replaced with fresh medium every 24 h. After incubation for 8 days, the biofilms were scraped off the glass coverslips with a sterile cell scraper, suspended in PBS and divided into aliquots for analysis. The biofilm biomass assay was conducted as proposed by Chunru [60].

To determine the dry weight, the bacterial suspension was transferred into a new preweighed centrifugation tube. Three volumes of 100% ethanol were added, and the mixture was incubated for 15 min at − 20 °C. The suspension was then centrifuged at 12,000 × *g* for 10 min. The resulting sediment was washed twice with 75% ethanol and lyophilized for 24 h. By subtracting the final weight from the initial weight of the empty tube, the weight of the biomass was measured, and the results are expressed in milligrams.

To obtain total soluble protein, 300 µl of bacterial suspension was transferred to a microcentrifuge tube, to which the same volume of 2 M NaOH was added. The tube was vortexed, placed at 100 °C for 15 min, and centrifuged (10,000 ×*g* for 10 min, 4 °C), and the concentration of soluble protein in the supernatant was determined by a BCA protein assay kit.

### Effect of LA/TCS@PLGA-NPs on virulence gene expression in *S. mutans* biofilms

Sterile cover slides were placed in sterile 6-well plates, to which 1.5 mL of *S. mutans* suspension was added in each well. After 24 h of incubation, 10 µL volumes of LA/TCS@PLGA-NPs (6 mg/mL), TCS@PLGA-NPs (4.5 mg/mL), TCS (0.88 mg/mL) and an equivalent volume of physiological saline were added individually to the wells. The plates were incubated for an additional 24 h. The biofilm was rinsed with physiological saline to remove free TCS and NPs, and the medium was replaced with fresh medium every 24 h. After 8 days of anaerobic culture, biofilm samples were obtained on the surface of glass coverslips, and RNA isolation was started.

Total RNA was extracted on ice using TRI Reagent according to the manufacturer’s instructions. The concentration and purity of the total RNA obtained were determined spectrophotometrically with a NanoDrop instrument (Thermo Scientific, USA). cDNA was synthesized with a PrimeScript RT reagent kit with gDNA Eraser. Real-time reverse transcription (RT)-quantitative PCR (qPCR) was performed using the SYBRPRIMES qPCR set. The primers used for qRT–PCR are listed in Additional file 1: Table S2. Real-time PCR detection was performed with a LineGene 9600 instrument (Bioer Technology Co. Ltd., Hangzhou, China) started from an initial denaturation at 95 °C for 30 s, and then 40 cycles of amplification, including denaturation at 95 °C for 10 s, followed by annealing and extension at 60 °C for 30 s, were performed.

The relative expression of each target gene was normalized to that of the internal control (the 16S rRNA gene of *S. mutans*). The melting curve profile was analyzed to evaluate the amplification specificity. The critical threshold cycle (CT) was defined as the cycle in which fluorescence could be detected above the background fluorescence and was inversely proportional to the logarithm of the initial number of template molecules. The data were analyzed by the instrument supporting software. Each assay was performed with at least three independent RNA samples in duplicate.

### LA/TCS@PLGA-NPs for dental caries in vivo

Female SD rats (17 days old) were purchased from Ensville Biotechnology Co., Ltd. (Chongqing, China). All procedures concerning animal experiments were approved and under the supervision of the Ethics Committee of Stomatological Hospital of Chongqing Medical University (2021–028). All rats were first fed a regular diet for 3 days to adapt to the new environment. Afterward, to prevent the effects of endogenous microorganisms, all rats were fed water containing 5% ampicillin and normal chow for 4 consecutive days. Then, the rats were randomly divided into 4 groups (n = 6), including the control group (sterile water), TCS group, TCS@PLGA-NPs group and LA/TCS@PLGA-NPs group. On Day 25, all rats in these groups were first subjected to 7 consecutive days of dental caries modeling. Briefly, after 16 h of culture, *S. mutans* cultures were centrifuged and diluted to 1 × 10^8^ CFU/mL in saline solution. A sterile cotton swab saturated with the *S. mutans* suspension was applied to the oral cavity of each rat for 15 s per quadrant, as described by Zhang et al. [61]. Then, oral swabs were collected and spread on MSA agar supplemented with bacitracin to confirm *S. mutans* colonization in the oral cavity. On Day 35, the rats in the four groups were treated with sterile water, TCS (0.88 mg/ml), TCS@PLGA-NPs (4.5 mg/ml) and LA/TCS@PLGA-NPs (6 mg/ml) twice daily for another 7 consecutive days. All rats were fed a cariogenic diet (Diet 2000#) supplemented with a 5% (w/v) sucrose solution from Day 25 until the experiment was finished.

After the rats were sacrificed by CO_2_ asphyxiation on Day 95, the jaws with teeth, blood and main organs were collected. The carious lesions were assessed via Keyes’ scoring. In addition, body weight monitoring, blood biochemical assays, routine blood examination and histological analysis of the main organs were performed to assess the biosafety of LA/TCS@PLGA-NPs in vivo.

Statistical analysis: At least three independent replications for each dataset of all experiments were performed, and the results are reported as the mean ± SD. Statistical analysis among multiple treatment groups was performed using GraphPad Prism via one-way ANOVA (Nonparametric). Statistical differences were considered significant at a value of *P* < 0.05.

## Conclusions

In summary, we developed a novel nanoparticle delivery system consisting of TCS-loaded PLGA nanoparticles to effectively interfere with *S. mutans* biofilms. The resulting LA/TCS@PLGA-NPs exhibited favorable properties, including a controllable size, negative charge, sustained drug-release kinetics and desirable safety profile. LA/TCS@PLGA-NPs can “coaggregate” with *S. mutans* depending on their “right-side-out”-oriented *lactobacillus* envelope and inhibit the formation of *S. mutans,* which was similar to inactivated *L. acidophilus*. The interaction between LA/TCS@PLGA-NPs and *S. mutans* was mainly related to the S-layer protein. In addition, LA/TCS@PLGA-NPs were mostly distributed in the medium layer and had a spatial affinity effect on *S. mutans* in the biofilm. These nanoparticles can continuously inhibit *S. mutans *biofilms through their sustained release ability and have a long-lasting inhibitory effect on the progression of caries in vivo. Overall, our work examined the characteristics of *Lactobacillus* envelope-coated nanoparticles and demonstrated their therapeutic potential against cariogenic biofilms. These nanoparticles may be considered feasible candidates for a new class of safe and effective drug delivery systems for the prevention of caries. Furthermore, this work provides new insights into cell membrane coating technology and presents a novel strategy to combat bacterial biofilms and associated infections.

## Supplementary Information

**Additional file 1: Figure S1.** Drug loading, encapsulation efficiency and drug release of LA/TCS@PLGA-NPs. (A) The loading efficiency and encapsulation efficiency of TCS@PLGA-NPs and LA/TCS@PLGA-NPs (n = 3). (B) In vitro drug release study with TCS@PLGA-NPs and LA/TCS@PLGA-NPs in PBS (n = 3). Data are presented as the mean ± SD. **Figure S2.** Cytotoxicity assay of LA/TCS@PLGA-NPs. HOK cell viability at various concentrations of (A) TCS, (B) PLGA-NPs, (C) TCS@PLGA-NPs and (D) LA/TCS@PLGA-NPs by CCK-8 assay. PLGA-NPs, TCS@PLGA-NPs and LA/TCS@PLGA-NPs at the highest concentration exhibited no obvious cytotoxicity on HOK cells. The data are presented as the mean ± SD. **Figure S3.** Analysis for the coaggregation of *Lactobacillus acidophilus* (ATCC4356) with *Streptococcus mutans* (UA159). (A) Quantitative analysis of coaggregation between *L. acidophilus* and *S. mutans* after 1.5, 3.5, 5 and 8 h of coincubation. (B) SEM images for the coaggregates of strains ATCC4356 and UA159 at 20,000 × magnification (bar represents 1 µm). The green arrow indicates *S. mutans*, and the red arrow indicates *L. acidophilus*. (C) CLSM images for the coaggregates of strains ATCC4356 and UA159 at 200 × magnification (bars represent 75 µm). **Figure S4.** Adhesion rate of LA/PLGA-NPs and PLGA-NPs to *S. mutans.* Data are presented as the mean ± SD. *P < 0.05. **Table S1.** Serum biochemical indices of rats in different groups. The data are expressed as the mean ± standard error of the mean. There was no significant difference in the indicators between groups (*P* > 0.05). **Table S2.** Nucleotide sequences of the primers.

## Data Availability

The datasets used and/or analysed during the current study are available from the corresponding author on reasonable request.

## Abbreviations

TCS: Triclosan
PLGA: Poly (lactic-co-glycolic acid)
CMCNPs: Cell membrane-coated nanoparticles
EPS: Extracellular polymeric substances
PAMAM: Poly (amido amine)
ALN-P: Alendronate-modified pluronics
CHX: Chlorhexidine
TEM: Transmission electron microscopy
DL: Drug loading
EE: Encapsulation efficiency
DMSO: Dimethyl sulfoxide
SDS–PAGE: Sodium dodecyl sulfonate-polyacrylamide gel electrophoresis
CCK-8: Cell Counting Kit-8
HOK: Human oral keratinocyte
FBS: Fetal bovine serum
DMEM: Dulbecco’s modified Eagle’s medium
CLSM: Laser confocal microscopy
