# Supplementary material for: Lactobacillus cell envelope-coated nanoparticles for antibiotic delivery against cariogenic biofilm and dental caries
Source: J Nanobiotechnology. 2022 Aug 2;20:356. doi: 10.1186/s12951-022-01563-x (PMC9344742; doi:10.1186/s12951-022-01563-x)
Supplement: Supplementary file 1 — Additional file 1: Figure S1. Drug loading, encapsulation efficiency and drug release of LA/TCS@PLGA-NPs. (A) The loading efficiency and encapsulation efficiency of TCS@PLGA-NPs and LA/TCS@PLGA-NPs (n = 3). (B) In vitro drug release study with TCS@PLGA-NPs and LA/TCS@PLGA-NPs in PBS (n = 3). Data are presented as the mean ± SD. Figure S2. Cytotoxicity assay of LA/TCS@PLGA-NPs. HOK cell viability at various concentrations of (A) TCS, (B) PLGA-NPs, (C) TCS@PLGA-NPs and (D) LA/TCS@PLGA-NPs by CCK-8 assay. PLGA-NPs, TCS@PLGA-NPs and LA/TCS@PLGA-NPs at the highest concentration exhibited no obvious cytotoxicity on HOK cells. The data are presented as the mean ± SD. Figure S3. Analysis for the coaggregation of Lactobacillus acidophilus (ATCC4356) with Streptococcus mutans (UA159). (A) Quantitative analysis of coaggregation between L. acidophilus and S. mutans after 1.5, 3.5, 5 and 8 h of coincubation. (B) SEM images for the coaggregates of strains ATCC4356 and UA159 at 20,000 × magnification (bar represents 1 µm). The green arrow indicates S. mutans, and the red arrow indicates L. acidophilus. (C) CLSM images for the coaggregates of strains ATCC4356 and UA159 at 200 × magnification (bars represent 75 µm). Figure S4. Adhesion rate of LA/PLGA-NPs and PLGA-NPs to S. mutans. Data are presented as the mean ± SD. *P < 0.05. Table S1. Serum biochemical indices of rats in different groups. The data are expressed as the mean ± standard error of the mean. There was no significant difference in the indicators between groups (P > 0.05). Table S2. Nucleotide sequences of the primers. [file 12951_2022_1563_MOESM1_ESM.docx]

**Additional file 1**

**Lactobacillus Cell Envelope-Coated Nanoparticles for Antibiotic Delivery against Cariogenic Biofilm and Dental Caries**

Luting Weng ^1, 2†^, Lang Wu ^1, 2†^, Rongjuan Guo ^1, 3†^, Jiajia Ye ^1, 3^, Wen Liang ^1, 3^, Wei Wu ^4,^^*^, Liang Chen ^1,*^, Deqin Yang ^1,*^

1. Stomatological Hospital of Chongqing Medical University, Chongqing 401147, China.

2. Chongqing Key Laboratory of Oral Diseases and Biomedical Sciences, Chongqing 401147, China.

3. Chongqing Municipal Key Laboratory of Oral Biomedical Engineering of Higher Education, Chongqing 401147, China.

4. Bioengineering College of Chongqing University, Chongqing, 400044, China.

^*^ Corresponding author.

† Luting Weng, Lang Wu and Rongjuan Guo contributed equally to this work.

**Corresponding** **authors:**

Name: Wei Wu

Address: Bioengineering College of Chongqing University, No.174 Shazhengjie, Shapingba, Chongqing, 400044, China.

Tel: +86 13883267463

E-mail: [david2015@cqu.edu.cn](mailto:david2015@cqu.edu.cn)

Name: Liang Chen

Address: Stomatological Hospital of Chongqing Medical University, No. 426, Songshi North Road, Yubei District, Chongqing 401147, China.

<Tel:+86> 13883877050

E-mail: chenliang@hospital.cqmu.edu.cn

Name: Deqin Yang

Address: Stomatological Hospital of Chongqing Medical University, No. 426, Songshi North Road, Yubei District, Chongqing 401147, China.

Tel: +86 18723257400

E-mail: [yangdeqin@hospital.cqmu.edu.cn](mailto:yangdeqin@hospital.cqmu.edu.cn)


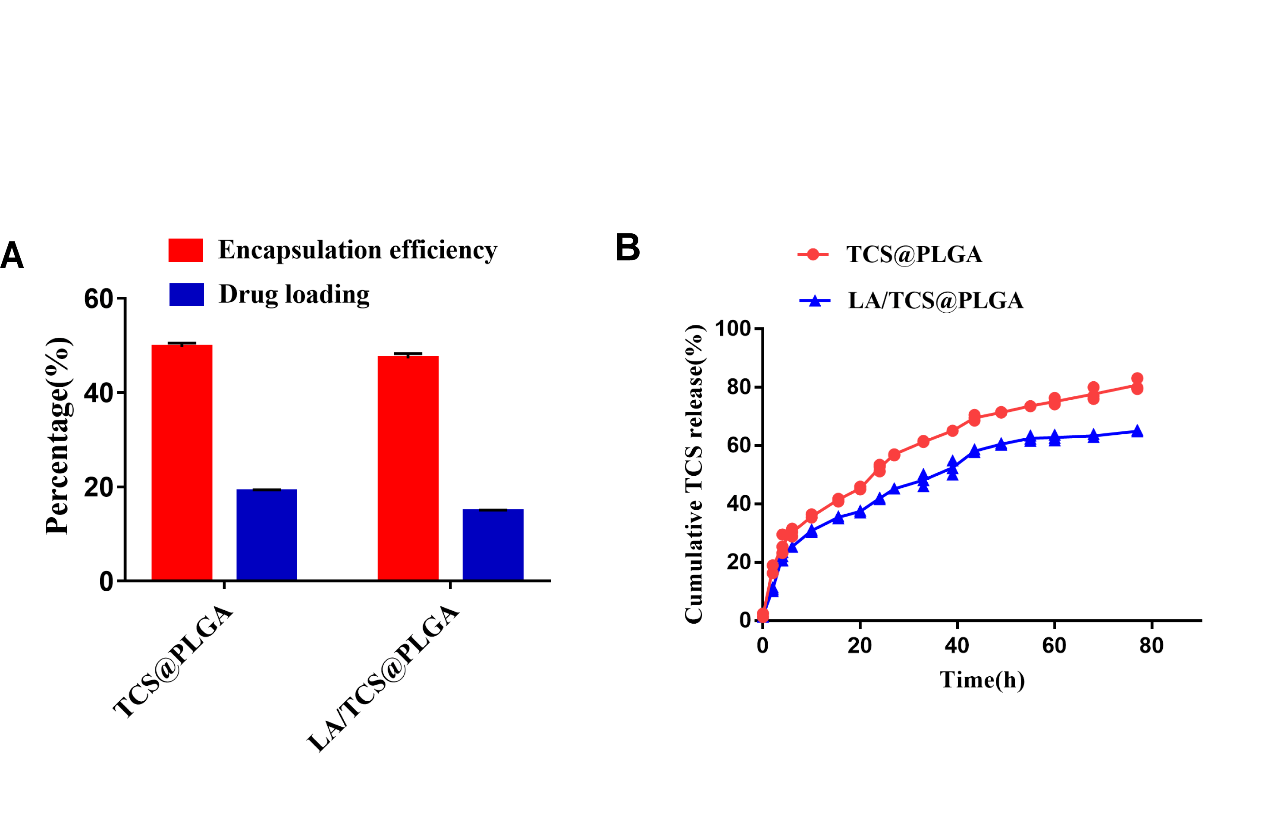


**Figure S1.** Drug loading, encapsulation efficiency and drug release of LA/TCS@PLGA-NPs. (A) The loading efficiency and encapsulation efficiency of TCS@PLGA-NPs and LA/TCS@PLGA-NPs (n=3). (B) In vitro drug release study with TCS@PLGA-NPs and LA/TCS@PLGA-NPs in PBS (n=3). Data are presented as the mean ± SD.


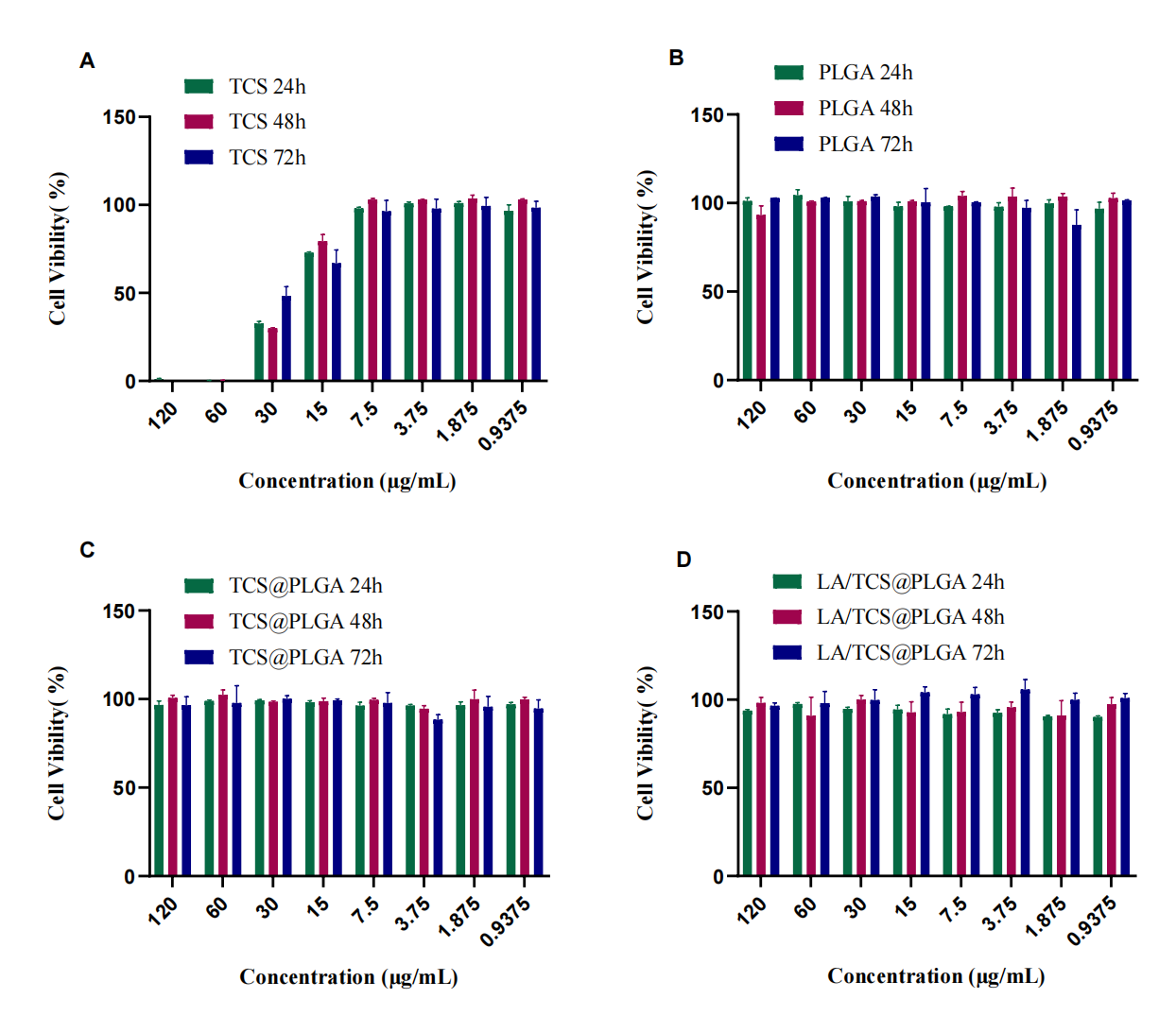
**Figure S2.** Cytotoxicity assay of LA/TCS@PLGA-NPs. HOK cell viability at various concentrations of (A) TCS, (B) PLGA-NPs, (C) TCS@PLGA-NPs and (D) LA/TCS@PLGA-NPs by CCK-8 assay. PLGA-NPs, TCS@PLGA-NPs and LA/TCS@PLGA-NPs at the highest concentration exhibited no obvious cytotoxicity on HOK cells. The data are presented as the mean ± SD.


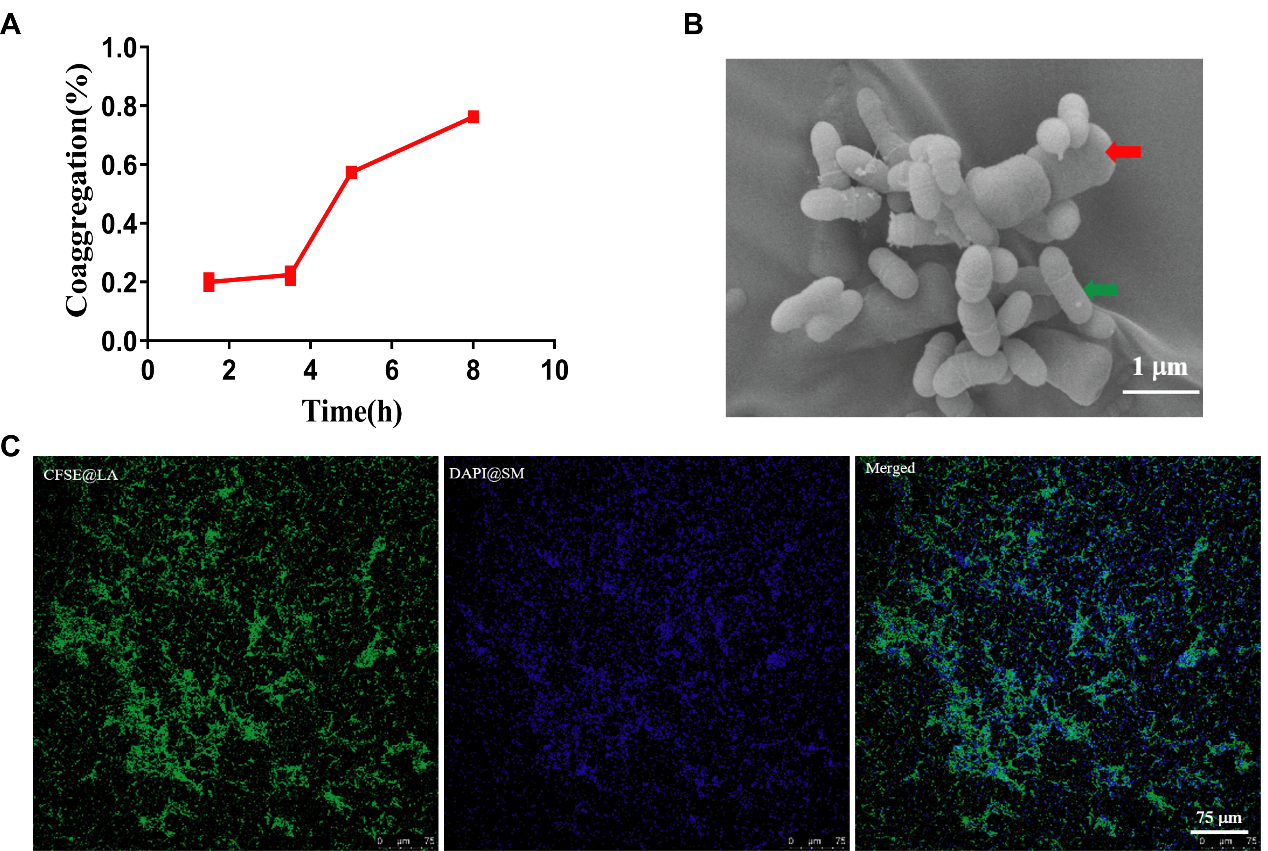


**Figure S3.** Analysis for the coaggregation of *Lactobacillus acidophilus* (ATCC4356) with *Streptococcus mutans* (UA159). (A) Quantitative analysis of coaggregation between *L. acidophilus* and *S. mutans* after 1.5, 3.5, 5 and 8 h of coincubation. (B) SEM images for the coaggregates of strains ATCC4356 and UA159 at 20,000 × magnification (bar represents 1 µm). The green arrow indicates *S. mutans*, and the red arrow indicates *L. acidophilus*. (C) CLSM images for the coaggregates of strains ATCC4356 and UA159 at 200 × magnification (bars represent 75 µm).


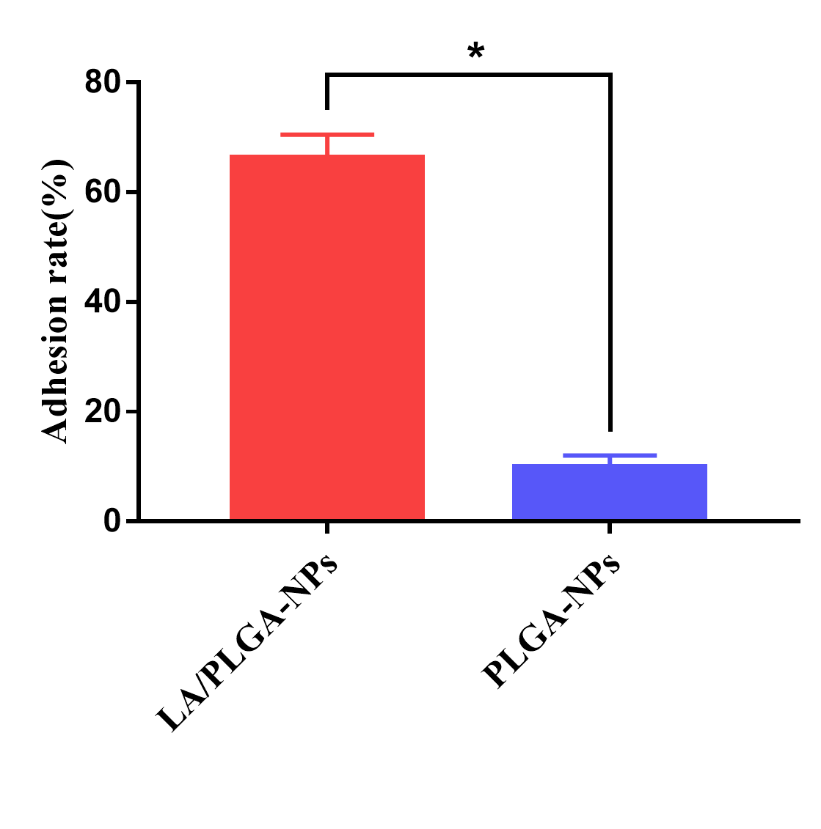


**Figure S4.** Adhesion rate of LA/PLGA-NPs and PLGA-NPs to *S. mutans.* Data are presented as the mean ± SD. **P* < 0.05.

**Table S1.** Serum biochemical indices of rats in different groups.

| Group | Biochemical index | | | | | | |
| --- | --- | --- | --- | --- | --- | --- | --- |
|  | TC | TG | HDL | LDL | IgM | IgG | Glu |
|  | (mmol/L) | (mmol/L) | (mmol/L) | (mmol/L) | (g/L) | (g/L) | (mmol/L) |
| NC | 2.02±0.09 | 1.24±0.18 | 1.43±0.01 | 0.28±0.01 | 0.31±0.04 | 0.74±0.01 | 18.51±0.48 |
| PC | 2.36±0.37 | 0.76±0.38 | 1.68±0.10 | 0.33±0.18 | 0.46±0.19 | 0.86±0.02 | 12.25±1.98 |
| TCS | 1.95±0.54 | 1.24±0.13 | 1.31±0.45 | 0.27±0.04 | 0.35±0.06 | 0.77±0.11 | 11.61±3.55 |
| TCS@PLGA-NPs | 2.15±0.37 | 1.47±0.73 | 1.50±0.18 | 0.27±0.03 | 0.33±0.20 | 0.83±0.04 | 14.14±3.83 |
| LA/TCS@PLGA-NPs | 1.86±0.05 | 0.67 | 1.30±0.03 | 0.27 | 0.33±0.03 | 0.82±0.01 | 11.20±0.71 |

The data are expressed as the mean ± standard error of the mean. There was no significant difference in the indicators between groups (*P* > 0.05).

**Table S2.** Nucleotide sequences of the primers.

| Primer name | Primer sequence (5’to 3’) | Gene description |
| --- | --- | --- |
| 16S-F | CCTACGGGAGGCAGCAGTAG | Normalizing internal standard |
| 16S-R | CAACAGAGCTTTACGATCCGAAA |  |
| ftf-F | CGAACGGCGACTTACTCTTAT | Fructosyltransferase (FTF) |
| ftf-R | TTACCTGCGACTTCATTACGATT |  |
| gbpB-F | AGCAGCGGCAGGATATAGAG | Glucan-binding proteins (GbpB) |
| gbpB-R | ACCAACCACGGTAGTTACCAATA |  |
| atpF-F | TTGATAACGCTAAGGAAACTGGTA | H^+^-translocating F-ATPase |
| atpF-R | AACGCTTGATAGGGCTTCTG |  |
| vicR-F | GCATCACTTAGCGACACACA | Two-component regulatory system regulator |
| vicR-R | CAGACGACGAACAGTAACATCAA |  |
| comD-F | ATGGTCTGCTGCCTGTTG | Com-dependent QS system |
| comD-R | CGATCATATAGGTGGTTA |  |
| gtfc-F | GTGACGACAAGTGAAGCAGCAAAAG | Glucosyltransferase SI (GTFC) |
| gtfc-R | AGTGGCGGTTGGTTGAGATGTTG |  |
| gtfb-F | TTAACTACACTTTCGGGTGGCTTGG | Glucosyltransferase B (GTFB) |
| gtfb-R | TCTTGCTTAGATGTCGCTTCGGTTG |  |
| spap-F | GGAGTGCGAGTAAGGAAGCTGAAC | Surface-associated protein P1 |
| spap-R | CATCGGCATCTTGGACAACATTGAC |  |
| ldh-F | ATATGAAGACTGTGCGGATGCTGAC | Surface-associated protein P1 |
| ldh-R | CCAACGAGATCGAGGCGAGTTTC |  |
